# Identification and expression profile analysis of odorant binding protein and chemosensory protein genes in *Bemisia tabaci* MED by head transcriptome

**DOI:** 10.1371/journal.pone.0171739

**Published:** 2017-02-06

**Authors:** Ran Wang, Fengqi Li, Wei Zhang, Xiaoman Zhang, Cheng Qu, Guillaume Tetreau, Lujuan Sun, Chen Luo, Jingjiang Zhou

**Affiliations:** 1 Institute of Plant and Environment Protection, Beijing Academy of Agriculture and Forestry Sciences, Beijing, China; 2 Laboratoire Intéraction Hôtes-Pathogènes-Environnement, Université de Perpignan, Perpignan, France; 3 Agricultural Information Institute, Chinese Academy of Agricultural Sciences, Beijing, China; 4 Department of Biological Chemistry and Crop Protection, Rothamsted Research, Harpenden, United Kingdom; Chinese Academy of Agricultural Sciences Institute of Plant Protection, CHINA

## Abstract

Odorant binding proteins (OBPs) and chemosensory proteins (CSPs) of arthropods are thought to be involved in chemical recognition which regulates pivotal behaviors including host choice, copulation and reproduction. In insects, OBPs and CSPs located mainly in the antenna but they have not been systematically characterized yet in *Bemisia tabaci* which is a cryptic species complex and could damage more than 600 plant species. In this study, among the 106,893 transcripts in the head assembly, 8 OBPs and 13 CSPs were identified in *B*. *tabaci* MED based on head transcriptomes of adults. Phylogenetic analyses were conducted to investigate the relationships of *B*. *tabaci* OBPs and CSPs with those from several other important Hemipteran species, and the motif-patterns between Hemiptera OBPs and CSPs were also compared by MEME. The expression profiles of the OBP and CSP genes in different tissues of *B*. *tabaci* MED adults were analyzed by real-time qPCR. Seven out of the 8 OBPs found in *B*. *tabaci* MED were highly expressed in the head. Conversely, only 4 CSPs were enriched in the head, while the other nine CSPs were specifically expressed in other tissues. Our findings pave the way for future research on chemical recognition of *B*. *tabaci* at the molecular level.

## Introduction

A sophisticated olfactory system with a pivotal role in many aspects of insect behavior, such as odorant detection, oviposition and mate recognition, is crucially important for insects’ survival and reproduction [1]. Insect olfactory-associated proteins mainly include odorant binding proteins (OBPs), chemosensory proteins (CSPs), odorant receptors (ORs) and sensory neuron membrane proteins (SNMPs). They are associated with diverse steps in the insect olfactory signal transduction pathway [2, 3]. Among these proteins, OBPs and CSPs are small soluble proteins that are abundant in the sensillum lymph of insect, especially in the head [4–6]. In the process of olfactory perception, these proteins exert a series of crucial roles in assisting insects for detection of chemical signals, therefore strongly affecting their behaviors [7]. As such, they are also significant molecular target candidates for the design and development of novel pest management strategies [8, 9].

The first description of OBP family in insect was for *Antheraea polyphemusin* in 1981 [10]. From then, a growing number of discoveries and identifications have been conducted, which highly benefited from the development and improvement of transcriptome and genome sequencing. Genome sequencing of *Acyrthosiphon pisum* identified 15 OBP genes [11]. On the other side, sequencing and analyses of transcriptome have revealed a large number of OBPs from diverse orders including notably Hemiptera, Lepidoptera, Hymenoptera and Coleoptera [12–20]. Typical OBPs protein sequence contains 6 conserved cysteines that form 3 disulphide bonds, whose role is to stabilize OBP tridimensional conformation. Furthermore, “non-classic” OBPs with various number of conserved cysteines have also been found and designated as Plus-C OBP (8 conserved cysteines), Minus-C OBP (4 conserved cysteines), Dimer OBP (12 conserved cysteines), and Atypical OBP (9–10 conserved cysteines) [5]. Based on qPCR analyses, it has become clear that many of the identified OBP genes are highly expressed in antennae [11, 13, 14, 21–23]. However, OBPs can also be highly expressed in other tissues which suggest that some OBPs might also be associated with taste perception and/or participate in other physiological functions [24, 25].

CSPs, another important family of carrier proteins, are also small soluble proteins and typically contain four conserved cysteines in their protein sequence with a similar role as for OBPs. Since the first identification of insect CSP in *Drosophila melanogaster* [26], numerous identifications of CSPs from many other species have been conducted. CSPs seem to be involved in the detection of common chemicals odorants and sex pheromones but can also be involved in other physiological and behavioral functions such as limb repair, growing development and feeding [27–31]. CSPs may play important role as carriers for molecule of odors through the sensillar lymph to transmembrane chemoreceptors. In addition to the heads of insects, some CSPs were also identified in other body parts such as thorax, abdomen, wings and legs in various insects [11, 32, 33].

The tobacco whitefly, *Bemisia tabaci* (Gennadius) (Hemiptera: Aleyrodidae) is now considered as a complex of genetically distinguishing cryptic species that often exhibit distinct host range and preferences, different symbionts community, and contrasting capacity for insecticide resistance development and for virus transmission [34]. *B*. *tabaci* is mainly distributed in the tropical and subtropical areas, and it is considered to be a highly cryptic species complex with more than 30 described species [34]. *B*. *tabaci* impairs plants directly by stylet probing and indirectly by acting as a vector for begomoviruses [35]. Within the species complex, the Middle East-Asia Minor1 (MEAM1, formerly known as biotype ‘B’) and the Mediterranean (MED, formerly known as biotype ‘Q’) species are highly invasive and have caused considerable economic damages to many important crops. After MEAM1 was first detected in China in the mid-1990s, it replaced the native whitefly species rapidly and became the dominant whitefly in both greenhouse and field crops [36, 37]. In 2003, MED was first detected in Yunnan province of China [37], and by 2007, MED had replaced MEAM1 as the dominant whitefly in China [38, 39]. Due to the extremely wide host range of *B*. *tabaci* MED, this species causes severe economic losses every year. At present, only 1 OBP and 5 CSPs cDNA sequences of *B*. *tabaci* have been published [40, 41]. To better understand the physiological mechanism underlying volatile detection in whitefly, more research on olfactory related proteins was required. Moreover, considering that these proteins can be key targets of pest control strategies, it is of high importance to extensively characterize OBPs and CSPs that may be involved in mating choice and host location in *B*. *tabaci*.

In the present study, we sequenced and analyzed *B*. *tabaci* the adult head transcriptome using Illumina sequencing. Then, OBPs and CSPs in *B*. *tabaci* MED were identified; sequence alignment and phylogenetic analysis were performed to characterize these molecules and quantitative real-time PCR was used to assess their expression in different tissues. In addition, the potential roles of the identified OBPs/CSPs transcripts in olfactory or other physiological processes were discussed. This work presents the first comprehensive characterization of OBPs and CSPs from the invasive agricultural pest *B*. *tabaci* MED, which may extend the list of molecular targets for *B*. *tabaci* control, give new insight into insect olfaction research and therefore provide an essential foundation for the development of better pest management strategies.

## Materials and methods

### Insects rearing and heads collection

*B*. *tabaci* MED were obtained from the Institute of Vegetables and Flowers in the Chinese Academy of Agricultural Sciences, and established in the laboratory at the Institute of Plant and Environment Protection, Beijing Academy of Agriculture and Forestry Sciences, China. All colonies were maintained on cotton plants (*Gossypium hirsutum* L. var. ‘Shiyuan 321’) under a 16 h: 8 h, light: dark photoperiod at 26±1°C and 70±10% humidity. Adult whiteflies were immobilized after incubation at 4°C for few minutes to separate males and females under a microscope (Nikon SMZ 1500), and then transferred to 1.5 mL centrifuge tubes for further dissection. Heads were excised from 2-day-old male and female adults (1000 heads for each sample), and promptly frozen and stored in liquid nitrogen until use.

### RNA isolation and sequencing library construction

Total RNA was extracted from male, female heads and other body parts of mixed-sex adults respectively using Trizol reagent (Invitrogen, Carlsbad, CA, USA) following manufacturer’s instructions. Quantity of RNA was determined on a NanoDrop ND-2000 spectrophotometer (NanoDrop products, Wilmington, DE, USA) and its integrity was verified by gel electrophoresis. cDNA library construction and Illumina sequencing of samples of the heads, female and male, respectively were performed at Shanghai Majorbio Bio-pharm Biotechnology Co. (Shanghai, China) using Illumina HiSeq 2500 (Illumina, San Diego, CA, USA). The mRNA was purified from 3 μg of total RNA using oligo (dT) magnetic beads and fragmented into short sequences in the presence of divalent cations at 94°C for 5 min. Then, the first-strand cDNA was generated using random hexamer-primed reverse transcription, followed by synthesis of the second-strand cDNA using RNaseH and DNA polymerase I. After the end repair and ligation of adaptors, the products were amplified by PCR and purified using the QIAquick PCR Purification Kit (Qiagen, Valencia, CA, USA) to create a cDNA library. Its quality was assessed on the Agilent Bioanalyzer 2100 system.

### De novo assembly of short reads and gene annotation

Clean short reads were obtained by removing those containing an adapter or poly-N and of low quality from the raw reads. Transcriptome *de novo* assembly was carried out with the short read assembling program Trinity (r20140413p1) by using default parameters [42, 43]. The resulting transcripts that were larger than 150 bp were first aligned by Blastx to protein databases, including NCBI Nr, Swiss-Prot, KEGG, and COG (E-value < 10^−5^), retrieving proteins with the highest sequence similarity for each transcript along with their protein functional annotations. Then, we used the Blast2GO program [44] to obtain a GO annotation of the transcripts, and GO functional classification with the WEGO software [45].

### Identification of OBP and CSP genes in *B*. *tabaci* MED

Similarity searches of OBPs and CSPs sequence were conducted using BLAST (http://blast.ncbi.nlm.nih.gov/blast.cgi), and the ORFs were predicted by using ORF Finder (http://www.ncbi.nlm.nih.gov/gorf/gorf.html). After cloning and sequencing, verification of putative OBP and CSP sequences was completed and steps are as follows. Template cDNA was synthesized using PrimeScript^™^ 1^st^ Strand cDNA Synthesis Kit (TaKaRa, Dalian, China) and PCR amplification was carried out in a Bio-Rad thermal cycler (Bio-Rad DNA Engine Peltier Thermal Cycler, Bio-Rad, USA) with the following thermal profiles: 94°C for 3 min; 35 cycles of 94°C for 30 s, 52°C for 30 s, and 72°C for 1 min, followed by incubation at 72°C for 10 min. The agarose gel electrophoresis was conducted with 1% agarose gels and DL2000 DNA Marker (TaKaRa, Dalian, China). We separated PCR products and purified the products of expected size with the Wizard DNA purification system (Promega, WI, USA). The DNA fragments from PCR amplification were cloned into a pMD18-T (TaKaRa, Dalian, China) and sequenced by Sunbiotech (Beijing, China). All candidate OBPs and CSPs were cloned and sequenced using the primers of validation (S1 Table). Putative N terminal signal peptides of BtabOBPs and BtabCSPs were predicted by Signal IP 4.1 (http://www.cbs.dtu.dk/services/SignalP/) [46]. We adopted nomenclature for the BtabOBPs and BtabCSPs that are analogous to those deposited in GenBank (http://www.ncbi.nlm.nih.gov/genbank/) and the rest of them were named based on their order in the head transcriptome data. Based on previous studies, BtabOBPs were divided into two groups: Classic OBPs, characterized by 6 cysteine residues at conserved positions and Minus-C OBPs, which are missing cysteine residues, generally C2 and C5 [5].

### Phylogenetic and motif analysis of the OBP and CSP genes

The phylogenetic trees were reconstructed for the analyses of BtabOBPs and BtabCSPs, using these genes (the signal peptides of sequences were removed from OBPs and CSPs) as well as sequences from other insects. The OBP dataset contained 8 sequences from *B*. *tabaci* and 72 from other hemipteran insects. The CSP dataset contained 13 sequences from *B*. *tabaci* and 53 from other Hemipteran species. The amino acid sequences of the genes used for phylogenetic tree construction are listed in supporting materials (S1 File). Amino acid sequences were aligned with Clustal X [47] and unrooted trees were constructed with MEGA5.1 [48] using the neighbor joining method, with Poisson correction of distances (OBPs and CSPs).

A total of 160 OBPs and 102 CSPs from different Hemiptera species were used for comparing the motif-pattern between Hemiptera OBPs and CSPs. All the OBP and CSP sequences used in this study are listed in supporting materials (S2 File) which have full ORFs and the translated proteins have similar length with insect OBPs and CSPs. The MEME (version 4.9.1) [49] on the line server (http://meme.nbcr.net/meme/), which has been widely used for the discovery of DNA and protein motifs, was used to discover and analyze the motifs in this analysis. The parameters used for motif discovery were as follows: minimum width = 6, maximum width = 10, and the maximum number of motifs to find = 8.

### Spatial expression analysis of *B*. *tabaci* MED OBPs and CSPs

RT-qPCR analysis was performed using gene-specific primers and SYBR Premix EX TaqTM (TaKaRa, Dalian, China) with three biological replicates in an ABI 7500 (Applied Biosystems, Foster City, CA, USA). Two housekeeping genes, β-actin (Accession number: EE600682) and EF-1α (AF071908), from *B*. *tabaci* were used as a reference [50] and 21 pairs primers for RT-qPCR were used (S2 Table). The RT-qPCR was carried out in 20 μl reactions containing 2 μl cDNA (200 ng/ul), 10 μl SYBR Premix Ex TaqTM (TaKaRa, Dalian, China), 1 μl forward primer (10 μM), 1 μl reverse primer (10 μM), 0.4 μl Rox Reference Dye II and 5.6 μl nuclease free water. Thermal cycling conditions were: 95°C for 30s, 40 cycles of 95°C for 5s, 62°C for 34s. After the cycling protocol, a melting curve analysis from 60°C to 95°C was applied to all reactions to verify a single PCR product. This was followed by the measurement of fluorescence during 55–95°C melting curve in order to detect a single gene-specific peak and to check the absence of primer dimer peaks. A single and discrete peak was detected for all primers tested. Negative controls were non template reactions (replacing cDNA with ddH2O). The results were analyzed using the ABI 7500 analysis software SDS1.4. Quantification of transcript level of all BtabOBPs and BtabCSPs was conducted in terms of the 2^-ΔΔCt^ method [51]. Expression levels of these genes were calculated relative to the two housekeeping genes using the Q-Gene method in Microsoft Excel-based software of Visual Basic [52, 53]. For each sample, three biological replications were performed with each biological replication measured in three technique replications. The comparative analysis of each target gene among various tissues were determined using a one-way tested analysis of variance (ANOVA), followed by Tukey’s honest significance difference (HSD) test using the SPSS Statistics 11.0 software (SPSS Inc., Chicago, IL, USA). The values were presented as the mean ± SE.

## Results

### Illumina sequencing, reads assembly and functional annotation

**Fig 1:**
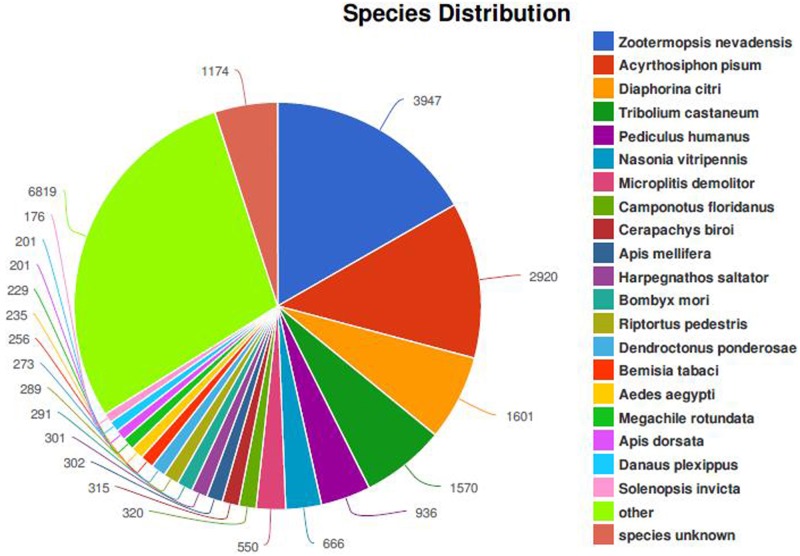
Percentage of homologous hits of the *B*. *tabaci* transcripts to other insect species. The *B*. *tabaci* transcripts were searched by BLASTx against the non-redundancy protein database with a cutoff E-value 10^−5^.

**Fig 2:**
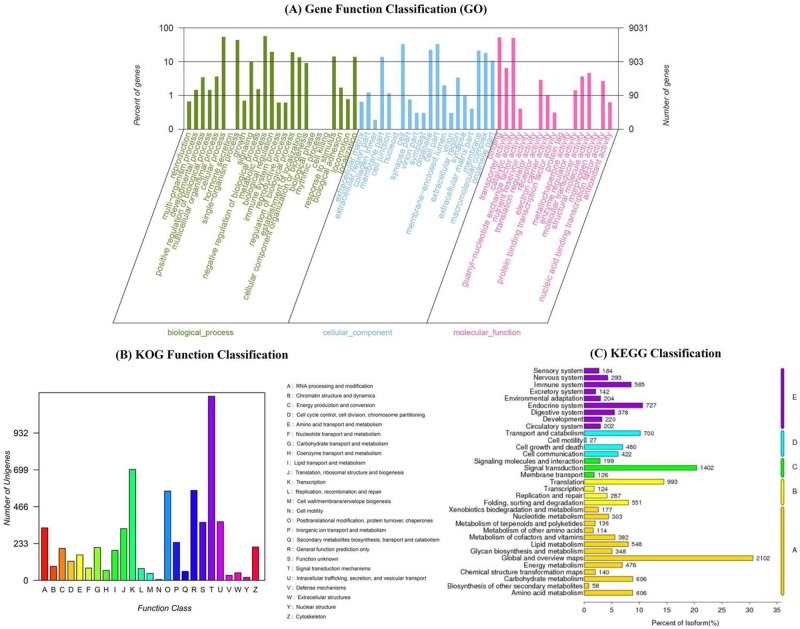
Analyses of head transcriptome of *B*. *tabaci* MED. (A) Gene Ontology (GO) analysis for the transcriptomic sequences. (B) Eukaryotic Ortholog Groups of proteins (KOG) annotation of the transcriptome. (C) Kyoto Encyclopedia of Genes and Genomes (KEGG) pathway annotation of the transcriptome.

To identify the OBP and CSP genes from *B*. *tabaci* MED, the cDNA from male and female heads were sequenced using the Illumina HiSeq 2500 platform. A total of 59,248,856 and 57,436,708 raw reads were obtained from male and female heads, respectively. After filtering out adaptor sequences, low quality sequences, 56,755,596 and 54,923,666 clean reads were generated from the heads of male and female raw data, respectively. Once assembled, 106,893 transcripts were obtained with an N50 of 2217 bp (Table 1). The raw reads of the *B*. *tabaci* MED were submitted to the GenBank Short Read Archive (SRA), under the accession number of SRX2403438 (female) and SRX2403439 (male). All the transcripts of the assembled transcriptome were blasted against nr datatbase (NCBI) using BLASTx and 23,572 transcripts were annotated, with an E-value below 10^−5^. Among the annotated transcripts, 25.4% had a best hit to Isopteran *Zootermopsis nevadensis* (dampwood termite) followed by 18.8% and 10.3% to the Hemipteran *A*. *pisum* (pea aphid) and *Diaphorina citri* (asian citrus psyllid), respectively (Fig 1). GO annotation was conducted to categorize the function of transcripts on the basis of the GO terms and a total of 9,031 transcripts were mapped to three GO groups (biological process, cellular component and molecular function) comprised of 50 terms (Fig 2A). In the terms of biological process, cellular and metabolic processes were the most ones. In the of cellular component category, cell and cell part were the highest classified. More importantly, the genes intensively expressed in the antennae were mostly associated with binding and catalytic activity, which were the most abundant in the molecular function terms. Moreover, 6,241 transcripts were classified to 25 KOG categories. Among these terms, ‘Signal transduction mechanisms’ (1164; 18.65%), ‘Transcription’ (702; 11.25%) and ‘General function prediction only’ (569; 9.12%) were the most common categories (Fig 2B). In the KEGG annotation, 11,179 transcripts were divided into five classes: cellular processes, environmental information processing, genetic information processing, metabolism and organismal systems. Among all pathways, several major ones were identified in each class including global and overview maps, translation, signal transduction, transport and catabolism, endocrine system (Fig 2C).

**Table 1:** Summary of assembled and annotations of transcripts.

	Transcripts	
Total sequence number	106893	
Total sequence base (bp)	105202375	
Percent GC:	39.25	
Smallest length (bp)	201	
Largest length (bp)	23032	
Average length (bp)	984	
N50	2217	
	**Number of Transcripts**	**Percentage (%)**
Annotated in NR	23572	22.05%
Annotated in Pfam	16473	15.41%
Annotated in String	7034	6.58%
Annotated in KEGG	11179	10.46%
Annotated in Swissprot	15471	14.47%
Annotated in all Databases	4085	3.82%
Annotated in at least one Database	4573	4.28%
Total Transcripts	106893	100

### Identification of OBPs and CSPs in *B*. *tabaci* MED

**Fig 3:**
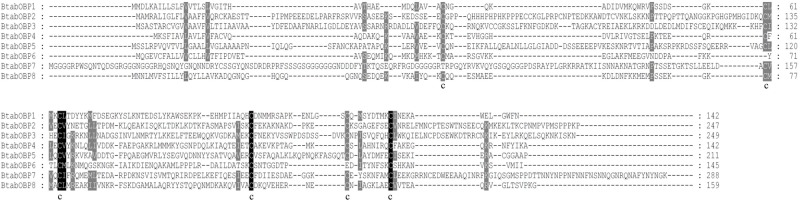
Alignment of the identified *B*. *tabaci* OBPs. Full-length amino acid sequences of *B*. *tabaci* MED OBPs are aligned by Clustal X 2.1. Black boxes show conserved cysteines. The conserved Cys residues are indicated.

**Fig 4:**
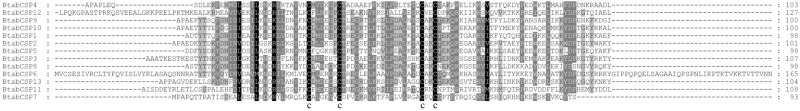
Alignment of the identified *B*. *tabaci* CSPs. Full-length amino acid sequences of *B*. *tabaci* MED CSPs are aligned by Clustal X 2.1. Black boxes show conserved cysteines. The conserved Cys residues are indicated.

Eight candidate OBP genes were identified from the *B*. *tabaci* MED Hiseq 2500 head transcriptome data (Table 2). All 8 OBP genes contained complete ORFs, which size ranged from 429 bp to 867 bp. All the full-length OBPs except one (BtabOBP7) contained a signal peptide at their N-terminal part, a signature of secretory proteins (Table 2). Based on the number and location of the conserved cysteines, the 8 full-length BtabOBPs could be divided into three families: BtabOBP6 belonged to the Minus-C OBP family, which have no conserved cysteines C2 and C5 while the remaining 7 BtabOBPs belonged to the Classic OBP family (Fig 3). Moreover, 13 different transcripts encoding candidate CSPs with four conserved cysteine profiles were obtained in *B*. *tabaci* through bioinformatic analysis (Fig 4), which were all full length and all except one (BtabCSP6) contained a signal peptide (Table 3).

**Table 2:** List of OBP genes in *B*. *tabaci* MED head transcriptome.

Gene name	Acc. No.	ORF (aa)	Signal Peptide	Full ORF	Best Blastx Match	Score	E value	% ID	FPKM	
									Female	Male
OBP1	KY305457	142	1–24	Yes	AHW57400.1 OBP1 [*Bemisia tabaci*]	271	1e-88	100%	638.393	251.113
OBP2	KY305458	247	1–22	Yes	AIS71883.1 odorant-binding protein 2 [*Bemisia tabaci*]	373	1e-118	100%	647.659	257.218
OBP3	KY305459	249	1–26	Yes	AGZ04907.1 odorant binding protein 7 [*Sogatella furcifera*]	178	2e-50	44%	116.169	67.57
OBP4	KY305460	142	1–19	Yes	AIS71884.1 odorant-binding protein 4 [*Bemisia tabaci*]	234	1e-74	100%	198.027	197.758
OBP5	KY305461	211	1–24	Yes	CAR85640.1 odorant-binding protein 4 [*Metopolophium dirhodum*]	172	6e-49	49%	0.372	0.526
OBP6	KY305462	145	1–25	Yes	XP_017882661.1 general odorant-binding protein 72-like [*Ceratina calcarata*]	69.7	4e-11	35%	112.878	174.996
OBP7	KY305463	288	No	Yes	XP_003244397.1 general odorant-binding protein 71 [Acyrthosiphon pisum]	143	9e-37	50%	10.824	8.894
OBP8	KT358500	159	1–21	Yes	AMD82868.1 odorant-binding protein 8 [*Bemisia tabaci*]	297	2e-99	100%	61.738	37.896

**Table 3:** List of CSP genes in *B*. *tabaci* MED head transcriptome.

Gene name	Acc. No.	ORF (aa)	Signal Peptide	Full ORF	Best Blastx Match	Score	E value	% ID	FPKM	
									Female	Male
CSP1	KT694344	126	1–19	Yes	ADG56568.1 chemosensory protein [*Bemisia tabaci*]	256	4e-85	100%	185.538	263.189
CSP2	KT694345	131	1–18	Yes	AEY84055.1 CSP1 [*Bemisia tabaci*]	266	2e-86	100%	2.028	3.372
CSP3	KT694346	138	1–20	Yes	AIT38537.1 chemosensory protein 3 [*Bemisia tabaci*]	224	3e-69	100%	331.063	508.891
CSP4	KT694347	128	1–19	Yes	ANJ43349.1 chemosensory protein 4 [*Bemisia tabaci*]	230	6e-74	100%	1430.187	1223.214
CSP5	KT694348	124	1–21	Yes	ANJ43350.1 chemosensory protein 5 [*Bemisia tabaci*]	207	1e-65	100%	418.504	234.613
CSP6	KY305449	246	No	Yes	AJP61956.1 chemosensory protein [*Phenacoccus solenopsis*]	131	3e-32	43%	4.271	10.631
CSP7	KY305450	112	1–19	Yes	ANA10244.1 chemosensory protein 2 [*Adelphocoris suturalis*]	155	3e-41	78%	33.127	31.634
CSP8	KY305451	123	1–18	Yes	SAJ59003.1 putative chemosensory protein [*Triatoma brasiliensis*]	176	1e-54	76%	0	0
CSP9	KY305452	127	1–19	Yes	AFJ54037.1 chemosensory protein [*Bemisia tabaci*]	117	6e-32	77%	0	0.493
CSP10	KY305453	127	1–19	Yes	AIT38553.1 chemosensory protein 1 [*Bemisia tabaci*]	86.3	9e-20	58%	0	0
CSP11	KY305454	136	1–18	Yes	AMA98180.1 chemosensory protein [*Blattella germanica*]	48.5	7e-05	33%	1.077	0.358
CSP12	KY305455	151	1–16	Yes	ACJ64045.1 putative chemosensory protein CSP2 [*Aphis gossypii*]	103	3e-23	40%	211.554	493.589
CSP13	KY305456	164	1–16	Yes	NP_001039289.1 chemosensory protein 7 precursor[*Tribolium castaneum*]	160	5e-43	60%	36.771	54.072

### Phylogenetic analysis of *B*. *tabaci* MED OBPs and CSPs

**Fig 5:**
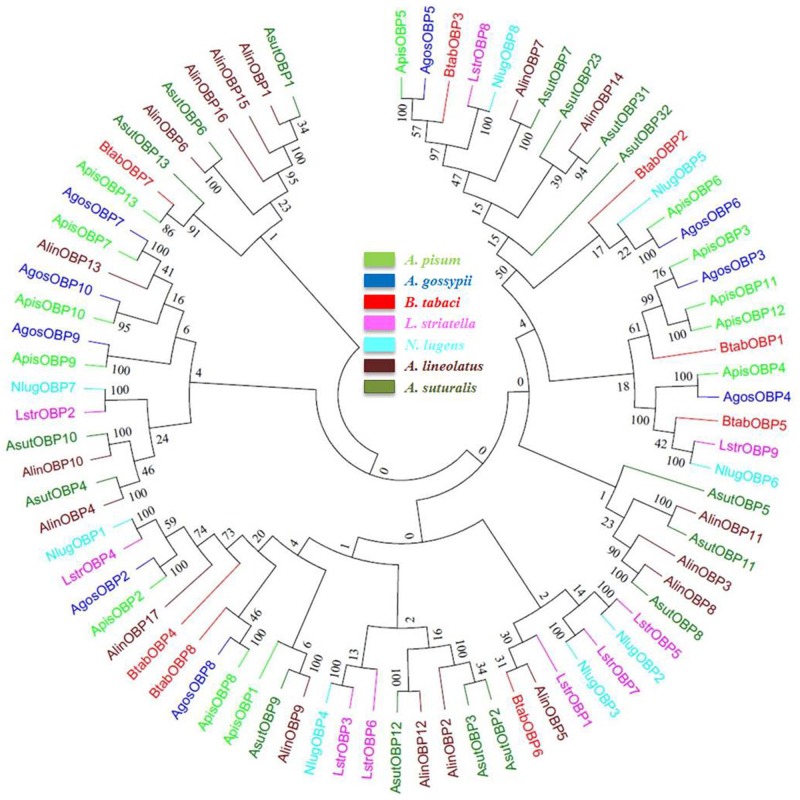
Phylogenetic relationship of candidate OBP proteins from *B*. *tabaci* MED and other hemipteran insects. The tree was constructed by MEGA 5.1 program using the neighbor-joining method with the Bootstrapping model by 1000 replication.

A phylogenetic tree of the OBPs was constructed with the 8 OBPs of *B*. *tabaci* and 72 OBP sequences of hemipteran pests such as *Acyrthosiphon pisum* (Apis), *Adelphocoris lineolatus* (Alin), *A*. *suturalis* (Asut), *Aphis gossypii* (Agos), *Laodelphax striatella* (Lstr) and *Nilaparvata lugens* (Nlug) (Fig 5). Although several sub-groups could be identified on the tree, the poor bootstrap support for the roots of most major sub-groups did not allow us to propose an OBP group naming nomenclature based on this phylogenetic analysis. In the neighbor-joining tree, none of the 8 BtabOBPs were clustered together as they were scattered among several different groups. BtabOBP2 clustered with OBP5 from *N*. *lugens* and two OBP6 from *A*. *pisum* and *A*. *gossypii* while BtabOBP3 clustered with the two OBP5 from *A*. *pisum* and *A*. *gossypii*. BtabOBP1 clustered with OBP3, 11 and 12 from *A*. *pisum* and OBP3 from *A*. *gossypii*. With a good bootstrap support, BtabOBP4 also clustered with a group of different OBPs from five different species including AgosOBP2 from *A*. *gossypii* which has been proved that it is very important in host selection and pheromone detection [54]. BtabOBP6 and BtabOBP7 were clustered together with AlinOBP5 and ApisOBP13, respectively. BtabOBP5 was clustered with NlugOBP6 and LstrOBP9, and BtabOBP8 was clustered with ApisOBP8 and AgosOBP8.

**Fig 6:**
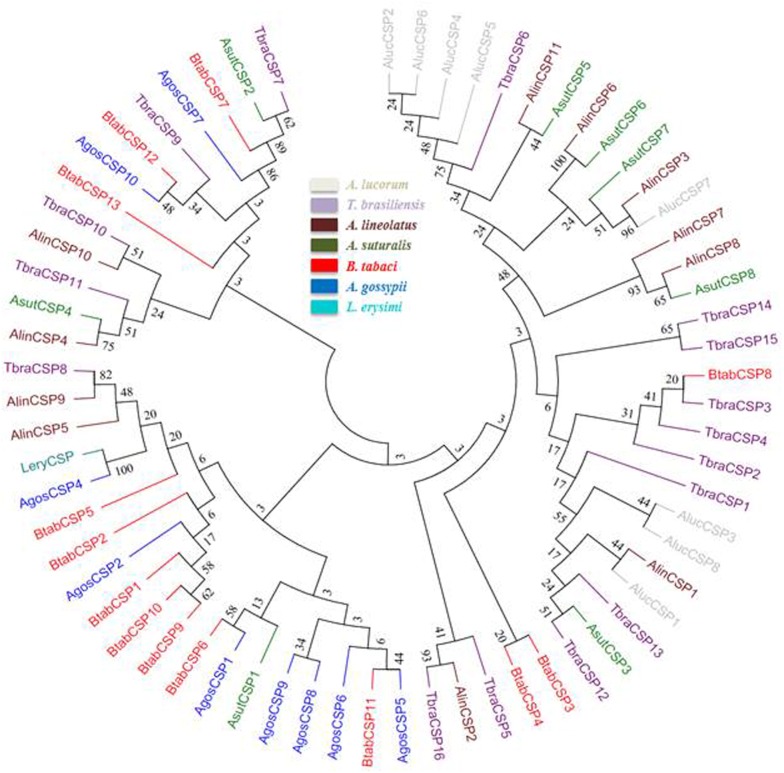
Phylogenetic relationship of candidate CSP proteins from *B*. *tabaci* MED and other hemipteran insects. The tree was constructed by MEGA 5.1 program using the neighbor-joining method with the Bootstrapping model by 1000 replication.

The neighbor-joining tree of the 66 CSP sequences, including the 13 from *B*. *tabaci*, was built from eight different Hemiptera species, including *A*. *lineolatus*, *A*. *suturalis*, *A*. *gossypii*, *Apolygus lucorum*, *B*. *tabaci*, *Lipaphis erysimi*, *Myzus persicae* and *Triatoma brasiliensis* (Fig 6). A similar low bootstrap support of most groups impeded us to propose nomenclature for CSPs. Nevertheless, contrary to OBPs, some CSPs from *B*. *tabaci* clustered together on the tree such as BtabCSP1, 9 and 10, and BtabCSP3 and BtabCSP4. All other BtabCSPs were clustered with CSPs from the other Hemiptera species. Among these Hemiptera species, some AlinCSPs from *A*. *lineolatus* may play important role in its host seeking [55].

### Motif pattern analysis of *B*. *tabaci* MED OBPs and CSPs

**Fig 7:**
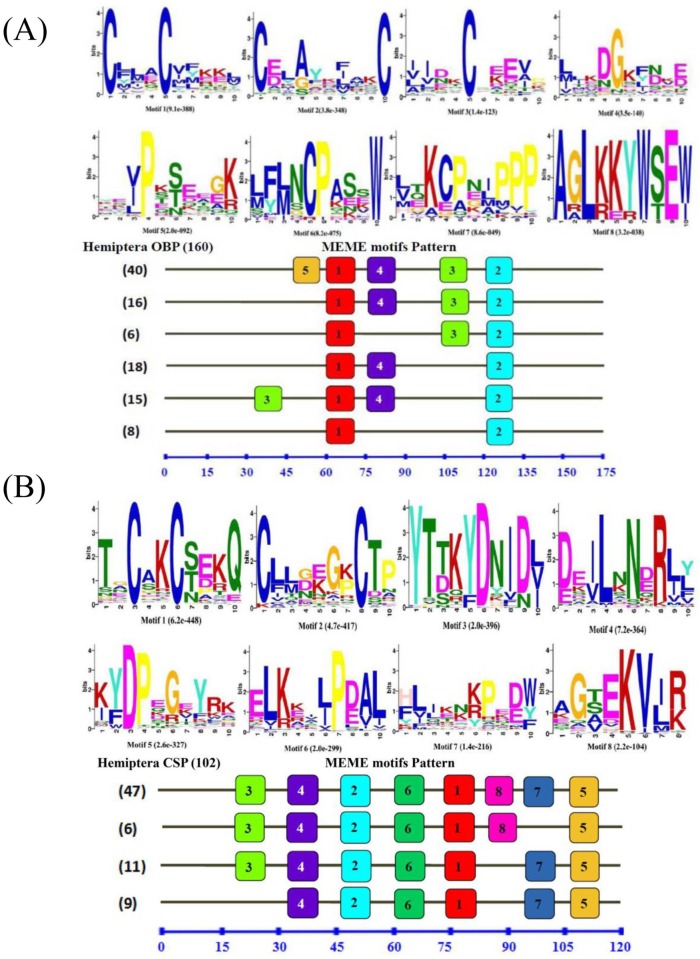
Motif analysis of Hemiptera OBPs and CSPs. Parameters used for motif discovery were: minimum width = 6, maximum width = 10, maximum number of motif to find = 8. The upper parts in (A, B) listed the eight motifs discovered in the Hemiptera OBPs and CSPs, receptively.

Besides, as most known OBP and CSP genes have been identified in the Lepidoptera [56, 57], we performed MEME motif analysis to compare the differences among several families of Hemiptera with 160 OBP and 102 CSP genes. When the motif-patterns of 160 OBPs from 13 Hemiptera species were compared, 23 different motif-patterns were found, among which 99 OBPs (61.9%) had the most common five motif-patterns (Fig 7A). Thirty-five of them had the same motif-pattern which is 5-1-3-4-2, 26 OBPs had the motif-pattern 5-1-3-2 and 16 OBPs had 4 motifs with the order 1-3-4-2. Moreover, 15 OBPs and 7 OBPs had only 3 motifs with the order 1-3-2 and 5-3-2 (Fig 7A). The remaining 61 OBPs shared the other 18 motif-patterns. On the other hand, the motif-patterns of the 102 Hemiptera CSPs were more conserved than the OBPs, 82 CSPs (80.4%) had the most common six motif-patterns, with 45 CSPs that had the motif-pattern sequence 3-4-2-6-1-8-7-5, 9 CSPs and 6 CSPs had 7 motifs with the order 3-4-2-6-1-7-5 and 3-4-2-6-1-8-5. Furthermore, 11 CSPs that had the motif-pattern 4-2-6-1-7-5, 6 CSPs and 5 CSPs had only 5 motifs with the order 4-2-6-1-7 and 4-2-6-1-5 (Fig 7B). The remaining 20 CSPs shared other 8 different motif-patterns.

### Expression levels of *B*. *tabaci* MED OBPs and CSPs

**Fig 8:**
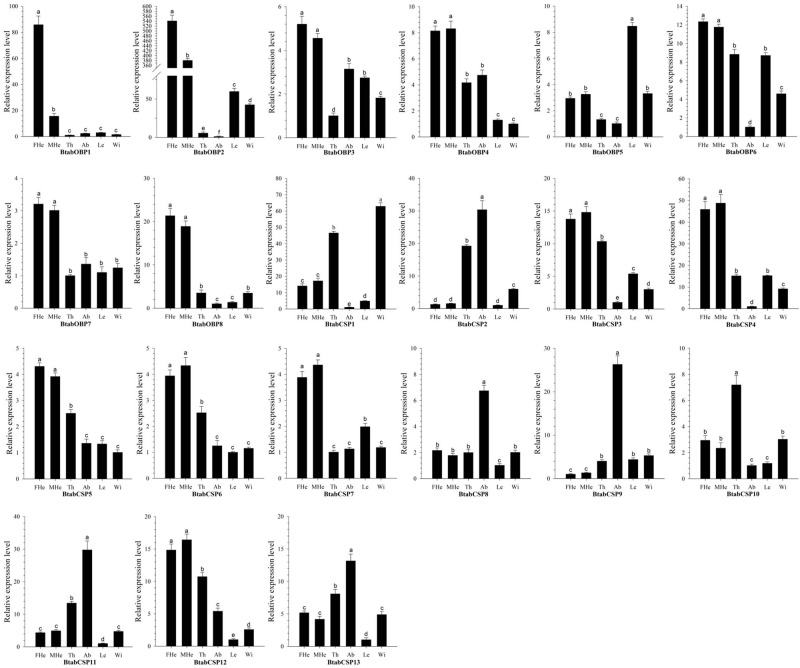
*B*. *tabaci* MED OBPs and CSPs transcript levels in different tissues as measured by RT-qPCR. FHe: female head; MHe: male head; Th: thorax; Ab: abdomen; Le: leg; Wi: wing. The expression levels were estimated using 2^- Δ ΔCt^ method. Standard error for each sample is represented by error bar and the different letters (a, b, c, d, e) above each bar denote significant differences (p < 0.05).

Real-time quantitative PCR (RT-qPCR) analyses were conducted to measure the expression levels of the 8 BtabOBP and 13 BtabCSP genes in the female heads, male heads and body parts (mixture of thorax, abdomen, legs and wings) (Fig 8). The results indicated that 13 of the 21 genes (BtabOBP1-4, BtabOBP6-8, BtabCSP3-7 and BtabCSP12) were approximately 3 to 500 times more expressed in both the male and female heads than in the other body parts (Fig 8). Furthermore, the two head-specific OBPs (BtabOBP1 and BtabOBP2) exhibited an expression level of 5.5 and 1.4 times higher in the female heads than in the male heads, respectively (p < 0.05). For CSPs, 5 BtabCSPs (BtabCSP2, BtabCSP8, BtabCSP9, BtabCSP11 and BtabCSP13) were mostly expressed in the abdomen (p < 0.05), with expression levels 2.5 to 26.1 times higher than in the heads. The remaining 3 of the 21 genes (BtabOBP5, BtabCSP1 and BtabCSP10) showed significant expression levels (2.56 to 8.33- fold, 1.35 to 61.94- fold, and 2.38 to 7.11- fold, respectively) in the legs, wings and thorax than in the other body parts (p < 0.05).

## Discussion

In the process of insect olfactory, OBPs and CSPs are responsible for capturing outside odorants and deliver them to the olfactory receptors, which are important for their survival and reproduction [1]. In this study, we sequenced, analyzed and then firstly reported the transcriptome of heads from *B*. *tabaci* MED. In whole gene ontology (GO) annotation of the transcriptome, similar to the antennal transcriptome of *Halyomorpha halys* [58], 21 biological processes, 19 cellular components and 12 molecular functions were categorized. Among the three groups, several parts of the group of biological processes were associated with olfaction of arthropod such as establishment of localization, localization, signaling and response to stimulus. In the group of cellular components, macromolecular complex, membrane and membrane part played a supporting role in the olfactory process. Ultimately, in the group of molecular functions, the parts of binding, transporter activity and molecular transducer activity were also contributed to insect olfaction. Based on the *B*. *tabaci* MED head transcriptome using Hiseq 2500 sequencing, we identified 8 OBP and 13 CSP genes from. The BtabOBP8 and BtabCSP1-5 have already been reported in previous studies [40, 41], but the other 7 BtabOBPs and 8 BatbCSPs are reported in the present study for the first time. The number of OBPs in *B*. *tabaci* MED is similar to that in some other Hemipteran species like in *A*. *gossypii* [14] and *N*. *lugens* [22] (with 9 and 10 OBP genes, respectively) but it is far lower than in *L*. *lineolaris* and in *A*. *lucorum*, which have 33 and 38 OBP genes, respectively [15, 21]. Concerning the number of CSPs, *B*. *tabaci* MED is relatively close to *A*. *gossypii* [14] and *A*. *pisum* [11], but contains much more CSPs than *Sitobion avenae* [32], which contain 9, 13 and 5 CSPs, respectively. For 8 BtabOBPs, 7 of them belong to the Classic OBP family, except BtabOBP6 which belongs to the Minus-C OBP family. In the 7 classic OBPs, BtabOBP7 had no conserved cysteines C1, which also occurred in SaveOBP13 from *Sitobion avenae* [32]. All 13 BtabCSPs had conserved cysteines C1–C4, which is typical of CSP and is shared by many other species [32, 41, 59]. Here, the 21 identified genes might belong to the three groups of the GO annotation and participate in a variety of chemical communications of *Bemisia tabaci* MED.

In the phylogenetic analysis, considering that the OBPs and CSPs do not form a species-specific cluster in the phylogenetic tree but rather form subgroups in which all species are mixed, it suggests that the diversity of OBPs and CSPs observed in each species appeared before the evolutionary radiation of the different Hemipteran species. For CSPs, we can see that some are clustering together, which suggests that there might have been a recent duplication within *B*. *tabaci* of a restricted set of CSPs genes that may be linked to their key function in the insect, yet to be characterized. Furthermore, based on the MEME motif analysis, both BtabOBPs and BtabCSPs presented several different motif patterns, with the most common one being 5-1-3-4-2 and 3-4-2-6-1-8-7-5, respectively. To our knowledge, this is the first report of motif differences in OBP and CSP genes in *B*. *tabaci*. In consistent with several reports [56, 57, 60], analysis of C-pattern (Figs 3 and 4) and motif pattern (Fig 7) illustrate that OBPs are more divergent than CSPs. Moreover, combine with some previous studies [56, 57], we found that the C-patterns are similar, yet the motif patterns are diverse among different Orders. We speculated that C-pattern is the central configuration of OBPs and CSPs with high conservation. By comparison, however, motif patterns regulate the functions of OBPs and CSPs, causing differences in binding of semiochemicals in various insect Orders.

Previously, many studies have reported the putative physiological functions of head-specific OBPs, especially antennae-specific, in detecting the general plant volatiles and sex pheromones [40, 61–63]. The functional study of head-specific or head-enriched OBPs contributes to elucidate the molecular and cellular mechanisms of chemical recognition and drives the development of a semiochemical-based pest management strategy like in aphids [8, 9]. In present research, the RT-qPCR results showed that 7 BtabOBPs and 6 BtabCSPs were specific or enriched in adult heads, indicating their putative function in chemical detection. Moreover, our study revealed that expression of BtabOBP1 and 2 was biased toward females’ head, with the expression levels 5.5 and 1.4 times significantly higher in the female heads than in the male heads, respectively. *B*. *tabaci* shows a haplo-diploid life cycle. The males, derived from unfertilized eggs, are haploid while females are diploid [64]. Males and females *B*. *tabaci* are different in several biological parameters such as longevity, size of body, symbionts, and ability of transmitting plant viruses [65–68]. By the comparative transcriptome analysis, Xie et al. (2012) revealed that more than 1,350 genes were differentially expressed between males and females *B*. *tabaci*, essentially over-expressed in females [69]. In *S*. *litura*, female antennae-biased expression of two general-odorant binding proteins (GOBPs) is consistent with their binding to the sex pheromones and general plant volatiles with different binding affinities [61]. Therefore, it is very important to further study the putative role of BtabOBP1 and 2. For other heads OBPs and CSPs (BtabOBP3-4, BtabOBP6-8, BtabCSP3-7 and BtabOBP12), based on their similar high expression levels in both sexes, we can speculate that they also have a potential function in recognition of semiochemicals. Eight OBPs and CSPs expressed in other parts of the body including in thorax, abdomen, legs and wings (BtabOBP5, BtabCSP8-11 and BtabCSP13) were also reported for the first time in *B*. *tabaci*, and their functions remain to be solved as well as the head-specific OBPs and CSPs. Our study provides a starting point to facilitate functional studies of these OBP genes at the molecular level both *in vivo* and *in vitro*. Moreover, our results not only pave the way to further investigate the molecular mechanisms of olfaction, but also provide insight and direction for the development of alternative pest management strategies as well.

## Supporting information

S1 Table PCR primers. (PDF)

S2 Table RT-qPCR primers. (PDF)

S1 File Sequences for phylogenetic analysis. (PDF)

S2 File Sequences for MEME analysis. (PDF)
