# Supplementary material for: Identification and expression profile analysis of odorant binding protein and chemosensory protein genes in Bemisia tabaci MED by head transcriptome
Source: PLoS One. 2017 Feb 6;12(2):e0171739. doi: 10.1371/journal.pone.0171739 (PMC5293548; doi:10.1371/journal.pone.0171739)
Supplement: S1 Table — (PDF) [file pone.0171739.s001.pdf]

S1 Table. PCR primers

Primer name	Sequence (5’-3’)	
BtabOBP1	Forward	CGGGATCCATGATGGATCTCAAAGCTATTTTGC
	Reverse	CCCTCGAGATTGAACCAGCCAAGCTCCC
BtabOBP2	Forward	CGGGATCCATGGCAATGCGTGCGCT
	Reverse	CCCTCGAGAGGTTTAGGCGGTGGGCTC
BtabOBP3	Forward	CGGTGAGTGTTGTCTGTCGT
	Reverse	GCTAGTGAATCTCCGTTGAG
BtabOBP4	Forward	CGGGATCCATGAAATCTTTCATTGCTGTTTTAGC
	Reverse	CCCTCGAGAGCTTTGATGTAAAAGTTGCGCT
BtabOBP5	Forward	GAGATGTCGTCCCTCCGTCC
	Reverse	CCCACGTCCTCGATTTGGTA
BtabOBP6	Forward	TTTACCTTTTTCTACCGTCC
	Reverse	TGCGTAAGTTAAGTTGCCTC
BtabOBP7	Forward	GTATGGGCGGAGGGGGTA
	Reverse	AGCTCATGCACTTGACTTCCTT
BtabOBP8	Forward	TGTGTCCCAGGATATTCATT
	Reverse	CTGTTGCTGATTTCGGTTTA
BtabCSP1	Forward	TAGTGACTTGTGAAGTGAAG
	Reverse	GGCGTTTGGAAGGTTGGGAT
BtabCSP2	Forward	GAGTGTTTTCGATCCGGTGT
	Reverse	CGAGTAGCTGGCTCATTTGC
BtabCSP3	Forward	CGGTGGGTTTAGTTTATCCT
	Reverse	TCTTCCTCGTGCTTCTCTT
BtabCSP4	Forward	AGTGCGCATTCCGTCATTCG
	Reverse	GCTGCCCACGGTGTTCTT
BtabCSP5	Forward	AATGAGCAAGTACGTGTTTG
	Reverse	GCTTTGGAATGGAGTAACG
BtabCSP6	Forward	TGTAGACGCTCCCACGGTGT

	Reverse	GAACGAACGGGTTTTCCTGC
BtabCSP7	Forward	AACTTTCTCAGTAACTCCGC
	Reverse	TGGGACATTCTGTTTCTTTA
BtabCSP8	Forward	TCAACAATATGTCGAGGACA
	Reverse	TCTGCCTGTAGACACCGTTT
BtabCSP9	Forward	CATGTATTTGTTTTCCGTGG
	Reverse	TGGTAACTTGTTTGGCTGCC
BtabCSP10	Forward	GAGCATCAGTTAGCATTGGA
	Reverse	ATCTACAGAGAGCCGAGGAA
BtabCSP11	Forward	TTAGTTCCGTCCGCGTGAAA
	Reverse	TTTTGACACTCAGACCGGGG
BtabCSP12	Forward	GACCGGAGTCAACTGACGTT
	Reverse	TCGACGCTTATTGCACAGGA
BtabCSP13	Forward	GTCGGTGGAAATCTCGCGTT
	Reverse	TTGAAACGGAAGGTGGGCAT
