# Supplementary material for: Identification and expression profile analysis of odorant binding protein and chemosensory protein genes in Bemisia tabaci MED by head transcriptome
Source: PLoS One. 2017 Feb 6;12(2):e0171739. doi: 10.1371/journal.pone.0171739 (PMC5293548; doi:10.1371/journal.pone.0171739)
Supplement: S2 Table — (PDF) [file pone.0171739.s002.pdf]

S2 Table. **RT-qPCR primers**

Primer name	Sequence (5'-3')	
BtabOBP1	Forward	AAGTGCTTGACGGATTATTAC
	Reverse	GCATCATATTATCGCAGTGT
BtabOBP2	Forward	CTCTTATTGGTCTATTTCTCGTT
	Reverse	CTTCTTCTTCTGGCATTGG
BtabOBP3	Forward	CTATCTCGGTTCAGTTCCA
	Reverse	TGTCTTTCCACTCGCTAT
BtabOBP4	Forward	GTTTCTTGGAGTGCGTTTA
	Reverse	TCATCATCATCAGCCTCTT
BtabOBP5	Forward	AAGTAAAGGCTGTGGATGA
	Reverse	CGAGTAATAGTTGTTGTCTTGA
BtabOBP6	Forward	GTAGCAATACAGGTGGAGA
	Reverse	ATGACACTCTTGACATTAGC
BtabOBP7	Forward	TCGAATCAGATGCAGAGGGTG
	Reverse	TATCCGGGGGACTCATTCCA
BtabOBP8	Forward	TGATGGCGTGTCTTATGA
	Reverse	CTGAGGTTGAGTGCTGTA
BtabCSP1	Forward	GTCCTCTCGGCCGATACCTA
	Reverse	AAGCGGTTTTCAAAGCGTCG
BtabCSP2	Forward	AAGTTCTCGTGGTGTTGT
	Reverse	GAGTAAGCAGGTGAGGTAG
BtabCSP3	Forward	AGTGCGCCAAGTGTACTGAA
	Reverse	ATTTGGCCTCCAATTTCGCC
BtabCSP4	Forward	TTCCAGACCTGACCGAAACG
	Reverse	CTCTTGTTGTCCGGGTCGAA
BtabCSP5	Forward	AAACGCCTCATCCGCAACTA
	Reverse	CTTGGCGCATTCCGTTTTCA
BtabCSP6	Forward	TGTTCGGAGAGCATAGTGCG
	Reverse	GCATGCACTCGACAAGCATC
BtabCSP7	Forward	CCGACGGCTTAAAACATT
	Reverse	AGGACCTTCTGGATCTGT
BtabCSP8	Forward	GACAGTAATCTTCTTGTGCTTA
	Reverse	ATCGTTGTTCAGAATCTCATC
BtabCSP9	Forward	ACTACTTCAACTGCCTCAT
	Reverse	CAATCTTCTTCTGTTTCTCTGT
BtabCSP10	Forward	TTTGTCGGCAGTTTCATC
	Reverse	GAGGAGCTTCTCGTTCTT
BtabCSP11	Forward	GTCCTTGCACTAACGAGGGG
	Reverse	AACTGTGCGCACTATCCTCC
BtabCSP12	Forward	TTGGTGGAATCAAGGTGCGT
	Reverse	GAGCTCTTGGTATTCCCTCGG
BtabCSP13	Forward	CGTGGACCGAGTTCTCAACA
	Reverse	TTCTGCTTCTCCGTGCACTT

BtabActin	Forward	TCTTCCAGCCATCCTTCTTG
	Reverse	CGGTGATTTCCTTCTGCATT
BtabEF-1α	Forward	TAGCCTTGTGCCAATTTCCG
	Reverse	CCTTCAGCATTACCGTCC
