# Supplementary material for: Identification and expression profile analysis of odorant binding protein and chemosensory protein genes in Bemisia tabaci MED by head transcriptome
Source: PLoS One. 2017 Feb 6;12(2):e0171739. doi: 10.1371/journal.pone.0171739 (PMC5293548; doi:10.1371/journal.pone.0171739)
Supplement: S1 File — (PDF) [file pone.0171739.s003.pdf]

S1 File. Sequences for phylogenetic analysis

The phylogenetic analysis of OBPs

>ApisOBP1
ESDQVPINSSAAVESCLLETNMTRDEFEDMLTSPNARELTILKSHAHKCMFGC
VMRKNHIVNDGVVSKEVLSKYVLNFYGRPDYKRRLIIKDVEHIVDVCAKKVA
DESETDECELAATLVTCIVLEANKAGLVDDPARQI
>ApisOBP2
SDPCNISTCYKSGTTKPPMAVTPTHLPVQSSSTQTSHPQTTYAKDHVHGSTTT
KSGVNATVTTASGASVNGTEPPAVVKSSAGVTGNSTTPKPTMTEGHVALKQK
LNTIAVKCKDELHAPQEIMALVSNTVVPQNEQQRCYLECVYKNLNLIKNNKF
SVEDGKAMARIRFANQPEEHKKAVTIIETCEKEAVIDPKTTEKCAAGRVIRNCF
VKNGEKINFFPKA
>ApisOBP3
RFTTEQIDYYGKACNASEDDLVVVKSYKVPTTETGKCLMKCMITKLGLLNDD
GSYNKTGMEAGLKKYWSEWSTEKIESINNKCYEEALLVSKEVVATCNYSYTV
MACLNKQLDLDKST
>ApisOBP4
QKQEPSGKCRAPDKAPLNLEIIINTCQEEIKSALLQEALDILNDGNVEQNTPNY
SSRSKREAEEDLTNEERRVAGCLLQCVYKKVKAVDETGFPVVDGLMKLYNEG
VQDRNYYIATLSAVRHCISIAQQLKQQQPSKSFDDGQTCDLAYEMFECVSEKI
EENCGVENKSNN
>ApisOBP5
DAGHHRRGKELLDTEDSDFFRCKQASRKSCCGPENAMKRFGDKDKVAADEC
YAQVAEKFATVTATTPKQDLFSAEAVKITKKKQFCLHECIGKKNNLLTEDGSLN
KTFIADYAMKSVFKEQWQKQVGQKALDKCLEETYIPWPAEDKENVCNPVYV
QFQHCLWLQYESNCPANKIKITKKCEKTRNRYRMQKSTSN
>ApisOBP6
MPNILPNLDSTWEKCFETFKQFKDKPETKEYKEMAHGKEPPCLFQCIFMQSGL
TTSDGKLNEDAITKKMSEGINNDEKWKSIWQNSLNKCFDDVKQEDKKQILIM
NTPAGRLMKCFLRDMYMSCPKNVWVESSECLNMKDLVQKCPEMPPPVFKSP
PKLI
>ApisOBP7
YLSEAAIKKTQQMLKTVCSKKHSVEEDVFTNIKKGIFPEDNNNIKCYFACNFK
TMQLINQKGVIDKKMFKDKMSMMAPPNVYKILLPVIEQCTGKDKGEELCQSS
YNVIKCAHSVDPKSLEFLPL
>ApisOBP8
ENNQQNGPSDRSATIFQSCIAETKLSGDALKGFRSMSIPKTQAEKCMMGCLM
RKVNVINKGKFSVEEATKVAQKYYGTNEAMMKKAKDLIDVCAKKAQSTTEE
CALAGIVTTCIVEEAQKAGLSGGPGSRSRRTVSPKFRRDAM
>ApisOBP9
DDADAKDKELMSKLFTVVFKCFKDADWGTCGEMITTKYDITQAKYKQCTC
HMACAGEELGMINASGQPEPAKFLEYVNKINNPDIKSQLQLIYDKCQNVKGS
EKCDLAEQFAICAFKESPALKERVSTLMEMLVKMKPKSK
>ApisOBP10

RPQPDEMEEIKRTLYNACAGKFPITEEIKNNAKNSIISDDPTFKCFLKCCFDEMS
MIDEDGIIDGDSLKAMAPDHIKPILEQVIPSCTKNVKQDGCEASFEFISCGIKLN
PLIVALLPL
>ApisOBP11
IFTTEQIDYYGKACNASEDDLIVLKSYKVPSTETGKCLMKCMITKLGLLNDDG
SYNKTGMEAGLKKYWSEWATEKIETINEKCYEEGNTATLLYHVAIYFTCVSGD
YSDVQLLIHCDGMFEQEVGSRQVNLKLLIMLKIGLSEPKR
>ApisOBP12
DDLVVVKSYKVPTTETGKCLMKCMITKLGLLNDDGSYNKTGMEAGLKKYW
SEWSTEKIESINNKCYEEGDTSTLLYHVVIYFTCVKGGSSDVQLLVHCDGMFE
QAVGSRQVNL
>ApisOBP13
MENGVTDFTHVSHGNTGSRSKNHRQGSYPSADYDSGQSNSNYNRQSLPTRR
YRRDDSNEKSKRQKAAVTGSNNRLLGNNRFRNMTKTGGNQPGKGTYLDKM
DACTIHCVFNQLEMLNSNSRPDKYSIVNIMTNQIKDVELKEFIQDSIDECFDTL
ELDSHNNKCEFSKNFAVCMENKAQRNCDDWDENLSANKINSAGLQDGTNQQ
DKRKGY
>AlinOBP1
DEQTNAMVAKAFNKCREEFPISDDEIGGVREKTTIPESHNAKCLMACMLREG
KMLRDGKYEKENALIMADVLNKDDPASADKAKQLVETCAGKVGTDAGGDE
CEFAYKMAVCAAEEAKKLGVRPPDF
>AlinOBP2
YQEQLKQTIRDCQDGKEVTDDELEEFTKPLIPRNREEKCIMACVMRTYNIISN
GHYDPKIAFGILKGILKDHPEKLNKIKEVMDHCGEDVPSHMDDECDLAGEIM
QCEVKYQKAMGMA
>AlinOBP3
ISKEYSARMIAAKEKCQKEFNVTDSVVEDFMKRNIKPESKSGKCMVHCIMEE
MGMIDDHKINTEQVKLGNKEKWDDPALVELANQVADTCDQEVFTEGRCKCL
VAVEYMMCLATHGDEVGLPHVDFEDSQDS
>AlinOBP4
GELPEEMKEMAQGLHDSCVEETGVDNGLIAPCAKGNFADDAKLRCYFKCVF
GNLGVISDEGELDAEAFGSILPDSMQELLPTIKSCGGTTGSDPCDLAMNFNKC
LQKADPVNFLVI
>AlinOBP5
AMSQAQMKQAMKTVRNMCIPKSGVDKEALAKMVNGEFDESDQKLKCYLG
CVLGMMQAVKNNKINLTMVRNQITKMLAPERGQRILAAFESCATVTGDDNC
GLAFRFAKCIYDTDKEAFIVP
>AlinOBP6
KELTDEQKEQIFAEIKNCMESTKLTDEEFESIMAKKELPTSIEGKCFTKCLMEK
MEYLEEGGKINVIAVQAGMEENMEKESEITKAKEVIQQCADSVPPEDSCEYAY
GISQCMYNKMKEAGISGS
>AlinOBP7
QQEDCKTAPAGWPRRPPQCCDLPFPLEGMKKEFGSCIRQIGNRQSSAVPTAQA
VRDARLCIEECVYKGLGFMDEHKLNKDQLLEQLKKGIADKKDWTKPMEGAV

KRCHETITKRETPQEAACQDSAHEFTHCAMRELFLNCPASEWNNNDECNLVK
SRMQACPNIPPPPPPPPQGFRGQGPPPQ
>AlinOBP8
VINKDYLEKVVTAKDKCLKEFNVDDSVVEDFIVKYNKPQSESGKCMVACFM
EERGMMKDGKTITEQVMLDNQEKWIAATHVNMGKEVIDTCDKEVPNEEND
KCDLAVDYMMCLVKRGDEAGLPKMDVAQLKH
>AlinOBP9
SQRTKQQPKSKTKESVVGATRPRDAKATECVNKVNANEEESASFFRKEIPETE
AGKCLLACYLEGKGLIVGGKISSSGAARVAARAYPNNRVKTGNVKHILSHCG
TIAGRESNNCEMAYKLADCTTTLSDKFRL
>AlinOBP10
QELPPPGDVKNKTVVFKNSFLRSAKYCSSIYETSTLAIMALLMSEKSDDQNGK
CFLNCMLQRYRLMSQDGSYNKDKFKPFLEYIPDSKFLQSIRGNLKNCISEKDP
DPCEKASKFIKCFYTRARNKGEIGASKEVIPADGF
>AlinOBP11
ISKEYHDKAIEAKNTCAKLHNVDDETIMTYWKNHQLPEKEPETCIVICYLKE
MKLVVDGKVDADAWKASNKEKWDDEKHVAAADEIVDKCSAEVPPTENECE
WGLALTKCALKHGKEAGIPPPDMEHPKRR
>AlinOBP12
MTTKLRSIGLVFIVSISYAFAYQELLKETIKKCQNGRDVTDDEVEEFTKPLVPK
NEEERCLVACVFKEYKVIIDGHFDPVNALNVAKVVYKDYPDKVERIKDVLDH
CGEDIPTHNDNECDLAGDIMKCEVKYLNSVPKMTSLEFLAGSMAATAEP
>AlinOBP13
LVSGHRALDGILPQANQDECREESNFRGELNDDVGRNVTQELKCFAACSLMK
LGIMNEKDGTVNMTRLDELIASHTPGKDAADVFKTTVVEPCMKEVKKSTDY
CEYSYQLIACGMSKVP
>AlinOBP14
MKPPGPPGSGTPPTAEERAARKIAHECADECLYKSSNLLTSAGELDKDAIKAL
VTKLYTGDWATAATTAIDKCLASAKGEVEATSKCKSGSFQLSRCFMRSMFLGC
PASSWTESTECAAAKARLTKCPNAMAPMPHKK
>AlinOBP15
MVDHWCASRIHKRYHELTHSRVVGGLRCCDEDEQTNAMVAKAFNKCREEFP
ISDDEIGGVREKTTIPESHNAKCLMACMLREGKMLRDGKYEKENALIMADVL
NKDDPASADKAKQLVETCAGKVGTDAGGDECEFAYKMAVCAAEEAKKLGV
RPPDF
>AlinOBP16
LKDGEYNKDNAELMADVLYKDEPEHAEKSKQIIELCSAELGTKTDGDDCEYA
YKMSVCAAKHAKELGVKTPEF
>AlinOBP17
VKEIVQNVSKKCAAETKASPEQAKIILTQNIPKNDVERCYLQCVYSGVGVIKD
GKFSQEGGNKLVAMRFHDAKEKELAKQLINTCAKEIKAKDGEKCSLGKGIRQ
CFVAHGKEVNFFPHA
>AsutOBP1
DEQTNAMVAKAFNKCREEFPISDDEIGGVREKTTIPESHNAKCLMACMLREG

KMLRDGKYEKENALIMADVLNKDDPASADKAKQLVETCAGKVGTDAGGDE
CEFAYKMAVCAAEEAKKLGVRPPDF
>AsutOBP2
YQEQLKQTIRDCQDGKEVTDDELEEFTKPLIPKNREEKCIMACVMRTYNIISN
GHYDPKIAFGILKGILKDHPEKLNKIKEVMDHCGEDVPSHMDDECDLAGEIM
QCEVKYQKAMGMA
>AsutOBP3
ELEEFTKPLIPKNREEKCIMACVMRTYNIISNGHYDPKIAFGILKGILKDHPEKL
NKIKEVMDHCGEDVPSHMDDECDLAGEIMQCEVKYQKAMGMA
>AsutOBP4
GELPEEMKEMAQGLHDSCVEETGVDNGLIAPCAKGNFADDAKLRCYFKCVF
GNLGVISDEGELDAEAFGSILPDSMQELLPTIKSCGGTTGSDPCDLAMNFNKC
LQKADPVNFMVI
>AsutOBP5
VNEGDASMEALMECKKDFKVSREQIMSGDSSEEVKCFAECLMKKTGGMDEG
GNFNTEKIKEEGRKHAKTDDQKRAHDAAVDKCISETEAANPTGKCEKGFEFF
KCVRGEMKSLM
>AsutOBP6
KELTDEQKEQIFAEIKNCMESTKLTDEEFESIMAKKELPTSKEGKCFTKCLMEK
MEYLEEGGKINVIAVQAGLEENMEKESEITKAKEIIQQCADTVPPEDSCEYAY
GISQCMYTKMKEAGISGGP
>AsutOBP7
QQEDCKTAPAGWPRRPPQCCDLPFPLEGMKKEFGSCIRQIGNRQSSAVPTAQA
VRDARLCIEECVYKGLGFMDEHKLNKDQLLEQLKKGIADKKDWTKPMEGAV
KKCHETITKRETPQEAACQDSAHEFTHCVMRELFLNCPASEWNNNDECNLVK
SRMQACPNIPPPPPPPPQGFRGQGPPPQ
>AsutOBP8
EGNINKEYLDKLIAAKEKCVKEFSVDDSIVEDLYVRYNKPPTESGKCMVACY
MEERGMMKDGKTITEQVMLDNQEKWIAATHVNMGKEVIDTCDKEVPNEEN
DKCDLAVDYMMCLVKRGDEAGLPKMDVAQLKH
>AsutOBP9
SQRTKQQPKSKTKENVVGATRPRDAKATECVNQVKANEEESASFFRKEIPETE
AGKCLLACYLEGKGLIVGGKISSSGAARLAARAYPNNRVKTGNVKHILSHCG
TIAGRESNNCEMAYKLADCTTTLSDKFKL
>AsutOBP10
QELPPPGDVKNKTVVFKNSFLRSAKYCSSIYETSTLAIMALLMSEKSDDQNGK
CFLNCMLQRYRLMSQDGSYNKDKFKPFLEYIPDSKFLQSIRGNLKNCISEKDP
DPCEKASKFVKCFYTRARNKGEIGASKEVIPADGF
>AsutOBP11
VSKEYHDKAIAAKNTCAKLHNVDDETIMKFWKAHQLPEKEPETCIIICYMKE
MKLVVDGKVDADAWKASNKEKWDDEKHVAAADEIVDKCSAEVPPTENECE
WGLALTKCALKHGKEAGIPPPDMEHPKRR
>AsutOBP12
MFQAFVYQELLKETIKKCQNGRDVTDDEVEEFTKPLVPKNEEERCLVACVFK

EYKVIIDGHFDPVNALNVAKVVYKDYPDKVERIKDVLDHCGEDIPSHNDNEC
DLAGDIMKCEVKYLNSIPKMTSLEFLAGSMAATAEP
>AsutOBP13
MGDVSNTYGSYDSYGASYGGYIRGSTPSGSGRGYKQSYEGDIGSGTSYARGA
GYGGAASYGTGYYNGNYGPDRTIYNPDSRTRGSYGGFMRGGGSSSNDGIFGA
TDFGSYAKQIDTTSYDSSESYDRVESYGGIPSGTPRGIPYNGYHNNANIRWPDS
NSQGNARKNGSSLEDVEPCTILCIFRQMKMTNGDSYLEQQSVAAVLMRRARD
PQLKDFIGRTVQMCFERFGLANKGRCESAKLFALCMEEAGKMNCEDWDVNK
RFALKNPKPGPVLTMPQPLPPPPPRG
>AsutOBP23
HPGHFDEDPECRPPHHHRLEEKDCCKIPNLFSNSRDEMHELVHKCFEEAGIKK
HGHHDHHGPPPPPGLGLPPPPPPPPKNDSKFDCVEQCFLKNLELINEEGEVKV
DELKALIAEKFTGDWASVGSSTIEKCLEKSKTEENDSSKCKAGSKRILICLARE
SFLSCPASEWTESDVCTAAKDRLEKCPHAPPPMNN
>AsutOBP31
MGRLTKTQNADLLIHPAGKIRVGVLPELFVGGIQDVIPCHDEARLKKPSGRHG
GGGSPPSAEDMAAHLRAHECADECVLKSQNLLTTDGELNKDAIKAQVVKSFT
GDWAKLASDTADKCLASAKGEVTATATCKSGAGQFIYCFRRNLFLQCPSSSW
TETTDCAAAKARITKCPNAKIPMGHHRH
>AsutOBP32
AECPQRFPKEMRPLSSCCTVEMNPNSSYNYTEGDPIINKCFGDFNSSATRPTGP
PSGYDCELECLMIEFGYMGKDKTINKDKIVKSIEDEYSADFQEAGHKAIEICM
GRKYHTSCPSGIDGMIECFAVQMMLNCPAKHWSGGEDCKETKQLIDKCGEVL
SIFADYDTD
>AgosOBP2
SDPCNISTCYKSGTTKPPTTVTPTRLPVQSSSTPTSHQQTTYAKDHVHSSTATK
SGVNTTATTTSGASVNGTERTTVVKSSSGVAGNVTTPKPTMTDGHVALKQKL
NTIAVKCKDELHAPQEIMALVSNTVVPQNEQQRCYLECVYKNLNLIKNNKFS
VDDGKAMAKIRFANQPEEHKKAVTIIETCEKEAIIDPKTTEKCAAGRVIRNCFV
KNGEKINFFPKA
>AgosOBP3
RFTTEQIDHYGKACNATEDDLVIVKSYKVPTSDTGKCLMKCMISKLGLLNDD
GSYNKTGMEAGLKKYWSEWSTDTIESINNKCYEEALLVSKDIIATCNYAYVV
MACLNKQLKLDNST
>AgosOBP4
QKQELSGKCRAPDKAPLNLEIIINICQEEIKSALLQEALDILNDGTLEQNTPSYS
RSKRDADEDLSNEERRVAGCLLQCVYKKVKAVDETGFPVVDGLMKLYNEGV
QDRNYYMATLSAVRHCISIAQQLKQQQPSKSFDDGQTCDLAYEMFECVSEKIE
ENCGVENKLNNLSQRQV
>AgosOBP5
EAGHQRRGKELLDTEDSDFFRCKQASRKSCCGPENAMKRFGDKDKVAADEC
YAQVAEKFATVTATTPKQDLFSGEAVKITKKKQFCLHECIGKKNKLLTEDGSLN
KTFIADYAMKSVFKEQWQKQIGQKALDKCLEETYIPWPAEETENKCNPVYVQ
FQHCLWLEYESNCPDNKIKLTKKCEKTRNRYRMQKSPSNQ

>AgosOBP6
VGFERTWILRQKRMTNDNECRALFPSPEKKLPTCCQMPNILPGLDNAWEVCF
EKFKQFKDKHATKEYKEMVHENEPPCLFQCVFMQSGLTTSDGKVNEDAVIKK
MAEGMDNDEKWKSIWRNTFNKCLNDVKQEDKEQIKVMNTPTGRLMKCFLR
DLYMNCPKNVWVENSECSNLKDLVEKCPKLPPPVFQSPPKLI
>AgosOBP7
YLSEEAIKKTQKMLKNVCSKKHSVEEEVFTDIKKGIFPENNNNIKCYFACNFK
TMQMVNQKGILDKKMFKDKMTMLAPPNVLAILLPPIEQCIGNDKDTEICQSS
YNFIKCAHRVDPKSLEFLPL
>AgosOBP8
ENNQQNSNDRSASIFQSCISETKLSGDALKGFRSMSIPKTQAEKCMMGCLMR
KVNVINKGKFSVEEATKVAQKYYGTNESMMKKAKDLIDVCAKKAQSTTEEC
ALAGIVTTCIVEEAQKAGLTGGPGSRSKRTVSPKFRHSIV
>AgosOBP9
ADDADTADKELMSKLITVAFKCFKDADWGTCGEMITTKYDITQAKYKQCTC
HMACAGEDLGLINSNGQPEPAKFLEYVKRINNSVIKSQLQHIYDKCQNVKGT
EKCDLAEQFAICAFKESPEMKERVTKLIEMLVKMKPKSK
>AgosOBP10
RPQPDEPDDIKKTLYNACSEKFPLTEEIKNNVKNSMVIDDQNFKCFLRCCFDE
MSLIDEDGIIDGESLAAMAVDKIKPVAEKIVHDCLPAGKQEKQDGCEAAFKFF
SCGMKLNPLTIELLPLQ
>BtabOBP1
VTHAEMDQLAVACNGQKADIDVMKQWRVPSSDSGKCLMKCLTDYYKMFDS
EGKYSLKNTEDSLYKAWSEKPKEHMPIIAQHCDNMMRSAPKENLGSCQMSY
DTMKCINEKAWELGWFN
>BtabOBP2
ESTTPIPMPEEEDELPARFRSRVVRSASEEKSKEDSSEECGPPQHHHPHPHKPPP
ECCKGLPRPCNPTEDKKAWDTCVNKLSKKNPTTPQPTTQANGGKPGHGPMH
GIDKQCMVECVYNETGLLTPDMKLQEAKISQKLTDKLKDTKFASMAPSVISK
CFEKAKNAKDPKECKSGAGEFSECVNRELFMNCPTESWTNSEECQKMKEKL
TKCPNMPVPMSPPPKP
>BtabOBP3
YDFEDAAFNARLIGDLDEYAVSSARNRRDADLDVDEFFQCKQRNQKVCCGK
SSLFKNFGDKDKTAGKACYREIAEKLKRKDDLDEDLMDIFSCEQIKQMKKKH
FCIHECIGKRKNLLNADGSINVLNMRTYLKKELFTEEWQQKVGDKAMEKCFN
EKYVSSWPDSDSSDVKCNPISVQFQHCLWKQIELNCPDSEWKDTRRCNKIKE
YLRKQELSQKNKISKN
>BtabOBP4
QDAKQKVAAVAEKCKTEVHGGHDVLRIVGTSELPKTEEQRCFLECVYKNLQL
IVDDKFAEPGAKRLMMMKYGSNPDQLKIAQTEIETCAKEVKPTAGMKCSLA
HNIRQCFAKEGQKRNFYIKA
>BtabOBP5
APNIQLQGSFANCKAPATAPQKLERVVEVCQNEIKFALLQEALNLLGLGIADD
DSSEEEEPVKESKNRTVTIAPAKSRPKRDSSFSQEERRVAGCLLQCVYRKVKA

VDDTGFPQAEGMVRLYSEGVQDNNYYSATVQAVERCVSFAQALKLKQPNQK
FASGQTCDLAYDMFECISEKIERACGGAAP
>BtabOBP6
AVSEQMIKQMKDMHKKTCMARSGVKKEGLAKFMEEGVNDDPAFKKYTLC
MLKNMQGSKNGKIAIKDIENQAKAMLPPPLRDAILDATSKCSNTGGDTPEDIT
YNFSKCSHKANVKSVMII
>BtabOBP7
MGGGGRPWSQNTQDSGRGGGNGGGRHQSNQYGNQNNDRYCSSGYQNSDR
DRPRFSSSGSGGGGGGGNDDDFYTKTQSEQRFRGDGGGGGRTRPGQYRVKV
QYGSGQGGGPDSRAYPLGRKRRATKIISNNAKNATGRNPTSSETGKTSLLEEL
DACVVQCIFRQMEMLTEDARPDKNSVISVMTQRIRDPELKEFIQESIEECFDIIE
SDAEGGKCEYSKNFAMCLEEKGRRNCEDWEEAAQINRFKGIQSGMSPPDTTN
NYNPPNFNNFNSNNQGNRQNAFYNYNGK
>BtabOBP8
DQGNQGHQGQQGNQSEDQEKVKAIYQKCQQESMAEEKDLDNFKKMEMPSS
EKGKCMMACLMREAKIIVNKRFSKDGAMALAQRYYSTQPQNMDKAKQVIV
ACDKQVEHERNECNIAGKLAECVVTEAQRVGLTSVPKG

>LstrOBP1
QIEQVGKAMANMCISSSGVQRSLITKAMKGEIEDDRKLKCFFGCIMEAVQVT
KNGRMQPEVLKRRANAMLPKTMREMILPTIDSCSHIENEDKCELAYSIVKCHF
SVNGKNPFFFNF
>LstrOBP2
RFSEEEKQLMNQVHAQCVTETGTAEDIVNKATKGDFTEDDNLKCYVKCIWST
LTVMDDEGDFDVGVLEVMLPADMKDIVMKAMSACTGAGAGLSPCEKAFAV
TKCLYKEAPADFFLP
>LstrOBP3
ANFAMMQQSLQGTNIPMIQSIAGELKFCMDVNSEQNSDGLDDYLPLLFNEEL
PTTLGQKCFLTCLFNRFGLLKDGFLDTQTAKTLVETFYKDKHDEKTMANIAIN
VCHVSAVPDVLNPCEIGFSLKSCFVDSNKKGKELRHKN
>LstrOBP4
DEATSSKPSSSPNAADALIASTTLSPASNETDAARAAIKEQLAKLTESCKTSSQ
ANSDEAKIIGTESVPKTEGEKCFLQCVYTGLGIVKNEQFSVEGAKLLAQKRFG
SFPEELEKANQLIETCSKEAVKKDSKDKCPMGFLIRQCFVKNGQKINFFPKA
>LstrOBP5
GLTPEKLKELKPLIDTCIKQSKAEEETIGKLQNGHEIPSSQTGKCFIACMAEHM
KLMKDGKFEPAMTMEFIDKMVQDKDKAAEIKKAVDDCFKSVPDTGDKCEM
AAGLATCMKEHHAELAGMN
>LstrOBP6
ADSSDMLTVFNKCRDKTSATEDDIKTFRAQQIPSTTTGKCMLACMFNHSGLM
KDGKYDSEGALKLVGQVFADNPIKLGKARQLINGCTDEVKKENDKCEIASKI
ADCTVKMSSQVGLS
>LstrOBP7
KIDKAKKEAAIKKCQAETSASDEDVKKVRKEHVVPESEEGKCFIACGFNTYD

MLKDNRINLEGVNAFFEKLYDEQEKRDIAIKAAASCAATESISGLNECHVAAK
YFACLQRHPDFVKMKEDFDV
>LstrOBP8
YDFSDPYFNEHLQSAMEEIMEEEMLSIGRVQRDADQGQEVADEYFKCKHRNL
KTCCGKINLMKNYGDKGKIYGKQCYEEVVSAFKSNSSSTADDDDSMMDMFS
CEKVKMIKLKHICVHECIGKKTKILKEDGSLNAEEIKQYAREYMFNEEWSKEL
GEKALDKCLTQTYNSVTKMLDEYEIKCNPTSVQFHHCLWKEIEMTCPESKVD
LKAKCVRLRERLRKQQAAGM
>LstrOBP9
EDTTIKIKNPSPHKQQQVYCQAPPTAPERLERIIEQCQDDIKTALLQEALNVLT
DTSPRDLVKKTRSKREVFSGEEKRIAGCLLQCVYRKVKAVDDQGMPTVPGLV
RLYSEGVQDRNYYVATVQAVQQCVSASQHFRYYNPQVLKEDGYTCDLAYDM
FNCVSDKIEAFCGRTP
>NlugOBP1
DEATSSSDAESLITSTTLSPASNESDAARSAIKEQLAKLTESCKTSSQANSDDAK
IIGTESVPKTEGEKCFLQCVYTGFGIVKNDQFSVEGARLLAQKRFGAFPEELEK
ANQLIETCSKEAVKKDSKDKCPMGFLIRQCFVKNGQKINFFPKA
>NlugOBP2
GLTPDKLKELKPLIDTCIKQSKVEEDTLGKLHNGHEIPSSQSGKCFIACMAEH
MKLMKDGKFEPEMTMEFIDKMVQDKDKAAEIKKSLGECIKSVPEGDKCEMA
AGLATCMKDHHAELAGMN
>NlugOBP3
KLDKAKKEAAIKKCQAETQATDEDVMKVRKEHIVPDSEEGKCFIACGFNSYD
MLKDNRINLEGVNAFFEKLYDEQDKRDIAIKAAASCAATETVSGLNECHYAA
KYFACMQRHPDFAKMKDDFDI
>NlugOBP4
ANFLMMQQSMQGTQMPMIQSIASELKFCMDVNAEQNSDGLNDYLPLLFNEE
LPSTLGQKCFLTCLFNRFGLLKDGFLDTKTAKNLVETFYADKHDEKTMANIAI
NVCHVAAVPDALNPCEIGFSLKSCFVDSNKKGKELRGKN
>NlugOBP5
VFIDGRNEYRLTRQAPPDDECRPPRPGPNEDGVCCDMPPVFRTAHDKFESCLE
ELSSIFPPPPPPPHGHHGPPPPPPGARGPPPPPPPGGRRGPPPGFGGPPGHEPPIFA
CAHECLFNKTGMLENGKLNVEALKKKLEDELGENEVWKNLVQSIVDKCMES
KDAPSNEMCTSGSHELARCVLRDMFMNCPQEKWKESDDCSNMKMKLEKCP
ELVPPMAMRLPHPPMP
>NlugOBP6
EDTTIKIKTPSPHKHQQVYCQAPPTAPERLERIIEQCQDDIKTALQEALNVLTDT
SPRDLVKKTRSKREVFSGEEKRIAGCLLQCVYRKVKAVDDQGMPTVPGLVRL
YSEGVQDRNYYVATVQAVQQCVSASQHFRYYNPQVLKEDGYTCDLAYDMFN
CVSDKIEAFCGRTP
>NlugOBP7
RFTEEEKQLMNQVHSQCISETGTSEDLVTKATTGDFADDDNLKCYVKCIWSTL
TVMDDEGNFDVGVLEVMLPADMKDTVMKAMNACTGVGGATPCEKAFAMT
KCLYKEAPSDFFLP

>NlugOBP8
YDFSDPYFNEHLQSAMEEIMEEEMLSIGRVQRDADQGQEVADEYFKCKHRNL
KTCCGKINLMKNYGDKGKIYGKQCYEEVVSAFKTNSSSTADDDDSMMDMFS
CEKVKMIKLKHICVHECIGKKTKILKEDGTLNPEEIKQYAREYMFNEEWSKEL
GEKALDKCLSQTYNSVTKMLDEYEIKCNPSSVQFHHCLWKEIELTCPESKVDL
KAKCVRLRERLRKQQAAGM

The phylogenetic analysis of CSPs
>AlinCSP1
AATYTSKYDNIDLDEILSNTRLYKKYFDCLANKGKCTPDGKELKESLPDALKT
NCAKCTKKQQEGTDKVFRHVLKNKPNDYKVLESIYDPPGIYRKKYEAEAEK
RGIKLPGSH
>AlinCSP2
AEVYTSKYDNIDVDKILSNDRILTRYIKCLMEEGNCTNEGKELKKTLPDALAS
GCTKCSEKQKAQTEKVLRHLSKNRPRDWALLKTKYDPKGEYSKKYEKEAKA
LTA
>AlinCSP3
AEQYTDKYDNIDIDEILNNDRMYKNYFHCVMGNGKCTPDGLELKAKIPEALQ
TECAKCTDKQKKEVEKVLRFIINQKKDDYKLLEEKFDPEGVYRKKYEAQKKL
VEEGKPIEY
>AlinCSP4
EMTEEEFYTKVFEEVDPDFILDNERILTSYLKCFYNEIECNAHAEVVKKSIPDV
LATVCGRCSDKQKSIFKYSLNKFIPAHPKDWEKILSIYDPSGEAWPKVKAFIES
>AlinCSP5
YTTHYDYIDVDQVLNNTRLYTKYVECLLGQGKCTPEARELRDKLPEALQTNC
ARCSERQASESHRVIRFLIQNRQEDFKLLEAKYDPSGLYFKRFEEETKRNVSLS
>AlinCSP6
ADKYTDKYDNIDIDEILTNERLYKKYFDCIQGTGKCTPDGIELKEKIPEALKTE
CAKCNEKQKAGVEKVMRYLITKKPEDFKILEDKFDPEGVYRKKYEAQRKLV
EEGKPVEY
>AlinCSP7
ASTYTDKYDNIDLDEILTNERLYKKYFDCIQGKGKCTPDGTELKEAIPDALKT
ECAKCNAKQKAGVEKVLRHLLTKKAEDYKILEDKFDPEGVYRKKYEAQKKL
ADEGKPIVL
>AlinCSP8
ADKYTDKYDNIDIDEILNNERLYKKYFDCILGNGKCTPDGTELKETIPDALKT
ACAKCNDKQKAGVEKVLRHLLTKKAEDYKILEAKFDPEGVYRKKYEAQKKL
AEEGKPIAL
>AlinCSP9
LAVVTREMREREFFRQLEVINVDSILINQRLIDKYIKCLLKTGKCDPIMKDLRI
ALPLILGHLCEARCSEK
>AlinCSP10
MRSNFINESIPDVLATVCGRCSDKQKSIFKYSLNKFIPAHPKDWEKILSIYDPSG
EAWPKVKAFIES

>AlinCSP11
KFFFSGLLLVCMASVSLCADEYTDKYDSVDLDEILNNQRLYQKYIDCVMGKG
KCTPDGALLKEKIPEAS
>AsutCSP1
QETYTSKYDNVNVEDALKNDRLYKAYFNCLADRGPCTREGNMLKEALPDGL
RNNCSLCTDPQRRGTHQVIRFLFKYRPEDMKLLEEIYDPEGIYKTKYAEERKK
LME
>AsutCSP2
AETRSSVSDEALEAALKDKRYLTRQLKCALGEGACDPVGRRLKTYAPLVLRG
ACPKCTPSEVRQIQQVLSHIQRHYPKEWAKILKQYAGQ
>AsutCSP3
ANQYTTKYDNIDLDDILKNQRLYKKYFECLTGKGKCTPDGKELKEHLPDALK
TGCSKCSEKQRAGSEKVIKHLLKNKPQDYAVLEKIYDPSGIYKKKYEAEAKKL
GINV
>AsutCSP4
EMTEEEFYTKVFEEVDPDFILDNERILTSYLKCFYSEIECNAHAEVVKKSIPDV
LATVCGRCSDKQKSIFKYSLNKFIPAHPKDWEKILSIYDPSGEAWPKVKAFIES
>AsutCSP5
GTYTDKYDNVNLDEVLNNERLYRNYFNCLQGKGKCTLDGAILKEVIPSALKT
DCALCSVRQKKGAEKVLIFLITKKPDDFKILEDKFDPEGVYRKKYEAQRKLVE
EGKPIH
>AsutCSP6
ADKYTDKYDNIDIDEILTNERLYKKYFDCIQGIGKCTPDGIELKEKIPEALKTEC
AKCNEKQKAGVEKVMRYLITKKPEDFKILEDKFDPEGVYRKKYEAQRKLVEE
GKPVEY
>AsutCSP7
SELYTDKYDSIDIDEILNNDRMYKNYFNCVMGNGKCTPDGTELKAKIPEALQ
TECAKCSDKQKKGVEKVLRFLIKEKKDDYKLLEEKFDPEGVYRKKYEAQKK
LVEEGKPIEY
>AsutCSP8
ADMYTDQYDNIDIEEILTNERLYKKYFDCIIGNGKCTPDGTELKETIPDALKTA
CAKCNDKQKAGVEKVLRHLLTKKAEDYKILEAKFDPEGVYRKKYEAQKKLA
EEGKPIVL
>AgosCSP1
MNILTIFCYVTVMCDTQVKPAVSAQRLQSVNQNVTPTNDGRKTIRETSSYPTR
YDYIDIEAVMNNERIIKILFNCVMSRGPCTREGLELKRIVPDAIQTECAKCNER
QRKQAGKVLAHLLQYKPEYWKMLVQKFDPNNVYLRKYMADNDDDEKLSL
QKLSNDTTKKKRNI
>AgosCSP2
AEEKYTTKFDNFDVDKVLNNNRILTSYIKCLLDEGNCTNEGRELKRVLPDALK
TDCSKCTDVQKDRSEKVIKFLIKNRSTDFDRLTAKYDPTGEYKKNLEKFEKER
ASAKPLKA
>AgosCSP4
APQKDAVAASGPAYTTKYDHIDVDQVLASKRLVNSYVQCLLDKKPCTPEGAE

LRKILPDALKTQCAKCNATQKNAALKVVDRLQKDYDAEWKQLLDKWDPKR
EHFQKFQQFLAEEKKKGFTKF
>AgosCSP5
SPAGTATAAAVSADDEIKDFPAYMKRFDKLNVEQVLNNDRVLASHLKCFLNE
GPCVQQSRDLKRVIPVIANNGCNGCTERQMTTIKKSLNFLRTKKPTEWARLVK
IYDPSGTKLNKFLDA
>AgosCSP6
APAKYTTKYDNVNIDEILNNDRLVASYFKCLMETGKCTPEGEEIKRWLPEAIE
NKCEDCSEKQKLGSEKIIKFLFEKKNDMWKQLEAKYDPQGTYRQRYAEEAK
KLNINV
>AgosCSP7
RPEDVKVENKPAVIKSETLAAPLPTNIVKRATDTIQLDSSLPNVSEDVLDKALS
DRRFVQRQLKCATGEGPCDPIGRKIKAHAPLVLRGMCVKCSQSEIKQIQRVMS
HIQKNYPKEYTKMLKQYQSGF
>AgosCSP8
ADGGIITPQQQQQQTMMFTAPTGYYVSTYDHIDVGRLLRNNKVVSGYVKCF
VNEGPCTPDGKLVKAYLLPEIIRTVCGKCTPRQKDMARMVLKHIYTYRQADF
EKIMQIYDTDGKRNEILAFMNH
>AgosCSP9
TFTRSTKFDDRTGIDIHLVKRDTDDVNDDENSVESDEGFFYRFTHFFQDSSDK
EDDDDDEKKPDFITTFDIFKLLDEEYAMQQFYCVINEDPCDEVGMRLKATIPE
EINRNCERCTSTERNNIRRILNYVKKHYPQFWKRVEPIYKKKI
>AgosCSP10
MINTRPRKLVRCIRGVSISVAKGDDAVNAENKDDDSHLVNREEIQRYMSMME
KINIDQMLNNTRLMSNNVKCFLNEGPCTAHLREMKKMVPMLVKDSCSSCTK
EQKIMMKKAMDAVKARRPNDYEKLSKFFDPEGKYEKKFLENLNESK
>AlucCSP1
AATYTSKYDNIDLDEILSNTRLYKKYFDCLANKGKCTPDGKELKESLPDALKT
NCAKCTKKQQEGTDKVLRHVLKNKPNDYKVLESIYDPTGIYRKKYEVEAEK
RGIKLPGSH
>AlucCSP2
ADKYTDKYDNIDVDEILGNQRLYQKYFDCIQGKGKCTPDGAELKKNIPEALQ
TDCAKCSEKQKAGVEKVLRHLINEKPEDYKVLEEQFDPEGVYRKKYEHLKK
KVEEGKPVEY
>AlucCSP3
AATYTTKYDNIDLDEILSNQRLYKKYYDCLANKGKCTPDGKELKEALPDALK
TNCSKCSKKQQEGTDKVLRYVLKNKPNDYKVLENIYDPSGNYRKRYEDEAS
KRGIKLPGSH
>AlucCSP4
SELYTDKYDNIDVDEILGNQRLYQKYFDCIQGKGKCTPDGAELKKNIPEALQT
DCAKCSEKQKAGVEKVLRHLINEKPEDYKVLEEQFDPEGVYRKKYEHLKKK
VEEGKPIEY
>AlucCSP5
AELYTDKYDNIDVDEILGNQRLYQKYFDCIQGKGKCTPDGAELKKNIPEALQT

DCAKCSEKQKAGVEKVLRHLINEKPEDYKVLEEQFDPEGVYRKKYEHLKKK
VEEGKPV
>AlucCSP6
SELYTDKYDNIDVDEILGNQRLYQKYFDCIQGKGKCTPDGAELKKNIPEALQT
DCAKCSEKQKAGVEKVLRHLINEKPEDYKVLEEQFDPEGVYRKKYEHLKKK
VEEGKPIEY
>AlucCSP7
AELYTDKYDNIDIDEILNNDRMYKNYFNCVMGNGKCTPDGLELKAKIPEALQ
TECAKCSDKQKKGAEKVLRFIINQKKDDYKLLEEKFDPEGVYRKKYEAQKK
LAEEGKPIEY
>AlucCSP8
TKYDNIDLDEILSNQRLYKKYYDCLANKGKCTPDGKELKEALPDALKTNCSK
CSKKQQEGTDKVLRYVLKNKPNDYKVLENIYDPSGNYRKRYEDEASKRGIKL
PGSH
>BtabCSP1
ADTYTTQFDNIDLEAILKNEKLVDNYTKCLMDEGPCTNEGRTLKKLLPDALK
TACAKCTEKQKTGARKVIKFYQTQHPEDFKKLQQKYDPEGKFKAEFEKALFG
QTL
>BtabCSP2
APAEDKYTDKYDNINVDDILGSKRLLKSYLTCLLDKSPCTPEGSELKRLLPDA
LKTACSKCTEKQKEGAARIVERVTAEYPTEWKELSAKWDPTGEYWAKYKPL
VQEYLKASA
>BtabCSP3
AAATKESTKESTYTNKYDNIDLGKILTNDRLFLNYFKCLMDEHTCSPDGAELK
KVLPDALSNKCAKCTERQRSGSEKVIRHLIDNKPEMWAKLEAKYDPKGTYR
KTYKNEAEKLGIKV
>BtabCSP4
APAPLEQSDLEKFENMDLSSILSNKRLRTAYVNCMVDKGPCTADAAEFKKILP
DLTETQCADCSAKFKELIKKSVSTFQKDYPEDWKTLMAHFDPDNKRAADLE
KFMSS
>BtabCSP5
DDFYSDKYDNIDLDSILASKRLIRNYMNCFQGKSPCTPEGTYLNQVLPEALKT
ECAKCTEKQREGAVKAIKKLSAEYPEEWKEITDKLDPTGEQYAKFKARFP
>BtabCSP6
MVCSESIVRCLTYFQVISLVYRLASAQSNNRATYTTKYDYINVDAVMKNERIL
KMLVECMLERGRCTREGLELKAAVPDALATDCAKCSQMQRKHASRVIAYLIT
YKKEYWNALATKYDPDGSYRRKYGIPPQPQELSAGAAIQPSPNLIKPTKTVKK
TVTTVNNTNNLFKAKKRMTREEKKEMVRRRFPQLHFMPNMWASNIQKIEKK
SERAPSIARRNVRRKAHTPTRRHSTQRTAPKRGQETG
>BtabCSP7
MPAPQTTRATISDEALESALNDKRYLMRQLKCALGEGVCDPVGRRLKTFAPL
VLRGACPQCSPTETRQIQKVLSHIQRHHPKEWSKIVKQFTS
>BtabCSP8
ASTYTTKYDNIDLDEILNNDRIYKKYYDCLANKGKCTPDGKELKDILPDALKT

GCSKCNEKQKKGSEKVIKFLLDKKKADYDVLEKIYDSNGVYRQKYADEAKK
R
>BtabCSP9
APAEFYTSQFDNIDIESILKNEKLLDNYFNCLMDEGPCTLEGRTLKSLLPDALN
TSCAKCTEKQKKIARRVMTFYLDKYPANSARIIKKYDPENKFKDGIEKALLGS
R
>BtabCSP10
APAETYTTEFDGIDIDSVLKNEKLLDAYAKCLLDEGPCTREGRTLKTLLPDALE
TTCAKCSPTQKEKAKKVITFYMEKYPENAQKIMKKYDPTGKYRKALEEAFL
GSL
>BtabCSP11
AISDDEYRLETLCSSPALEHFDITPILKNDRLVSSYFKCFMDEGPCTNEGKMVK
RIIPEIMRTQCRNCNPTMRRIVRTVMKHMFQTRPRDVDDFFLKYDPHEMYYD
DLIEFMDEDNDY
>BtabCSP12
LPQKGPASTPRKQSVEEALGKKPEELPKTMKEALKRMEAVDVEKVLNNDRIL
TNYLKCFLNKGPCTSEAKNVKKSIALLVESRCVECDPKQRKIIKKSMQVVKTK
KPREYQELIKLYDPKGTQIAELEKFFASSK
>BtabCSP13
APPAGVDEKLLSKYDNFDVDRVLNNDRVLANYIKCLMDEGSCTNEGRDLKK
SIPDVLAGGCDKCTEKQKSVTEKVIKHLINKRPKDWDRLSKKYDPQGQYKN
KYADLYEKVQKEAAKESKEPSKPTKDTKETTKVTKDTKESAKAPKA
>LeryCSP
APQKDTAVVNGPAYTTKYDNIDIDQVLASKRLVNSYVQCLLDKKPCTPEGAE
LRKILPDALKTQCTKCNATQKNAALKVVDRLQRDYDKEWKQLLDKWDPKR
EYFQKFQQYLAEEKKKGVVKF
>TbraCSP1
SSKYTTSYDNLDLDEILNNHRLYTRYFQCLTNKARCTPDGKELKAVLPDALAT
GCAKCTEKQKQGSEKVIRFLLKNKPGDYEELEKMYDPNGTYRHRYEEEAKK
LGIKV
>TbraCSP2
TSTYTTQYDNIDLDEILNNDRIYKKYFDCLAHNIKCTPDGKQLRDILPDALKT
ACSKCSEKQKKGSEKVIKFMLEKKRADFDVLESQFDPTGIYRKKYAEEAKKH
GVHI
>TbraCSP3
TYTTKYDNIDVEEILNNDRLYKKYYDCLANKANCTPDGKELKDILPDALKTQ
CAKCSEKQKKGAEKVVRFMLENKRADFDALEKIYDPSGTYRKKYAAEAKRR
GINV
>TbraCSP4
ASTYTTQYDNIDLDEILNHDRVYKKYFDCLVNKAKCTPDGKELKDILPDALK
TGCTKCNEKQKKGAEKVIRFLLENKRADFDALEKIYDPSGTYRKKYAPEAQK
YGINV
>TbraCSP5
TTYTTKYDNIDLDAVLRNDRVYKTYYNCLTNKGKCSPEGKELKDKLPDALQT

GCSKCSARQKQGLEKIIKFLMNSKQADFKEFERIYDPTGEYRKRYAEQAKKK
GIKV
>TbraCSP6
AKQSKVTYTDKYDKIDVDAILHNERVLKRYIDCLMDRARCTPDGAELKKYIP
EALETECAKCTEAQKRFAGKVMSFLLQNKRNYWNELLGKYDPNGKFRKKYE
EAALQDEDYSSFYNE
>TbraCSP7
AEGRSAVSDEALEAALKDKRYLARQLKCALGEGACDPVGRRLKTYAPLVLR
GACPKCTPSEVKQIQQVLAHIQRHYPKEWSKILKQYAGQ
>TbraCSP8
LTLQQIHRSWFQRLSVIPVEDVLNNRRILNKYLGCLLRRTVCAPEARDFRILLP
SILREPCNNCTERQRSSLKKIFEHVHTVHPGEWQEIMSMYDPKSEHQEKIINFI
SNS
>TbraCSP9
QYEVNVEEILNNKRLLDAYTKCYLDRGPCPGPARESKKKLGEVFTTNCAKCN
KKQRQDTRAALRKLRERKPQLFLEIFEKYDAGSKHLDGFLIWLKKND
>TbraCSP10
EEPDNEFNRLFDPNVDVDAVLDNDRVLNAYLACFYDEGPCAERPKLVKSKIR
EVLETTCGKCNDQQRQRLKYILNKFIDKRPNDWQRILEIYDPDGAFRDNVEK
LKQGLPAQ
>TbraCSP11
RPDSEEDAFYYQVFEEIDVDIILDNERLLRSYLTCFFDEAPCSAHAAAVKESIPE
VMSTVCGKCNDKQKAIYKHALNKFIPTHKEDWDHILRIYDPNGEYWPNIKKF
MES
>TbraCSP12
STYTTKYDNIDLDEILSNERIYVKYYNCLLNKSRCTPDGKELKENLPDALQTA
CDKCSEKQKQGSEKVLRFILEHRPQDYLALEEMYDPQKVYRHKYEKDAKER
GLKFPD
>TbraCSP13
ASTYTTQYDNIDLDEILNNERVYIKYFNCLIGKDQCTPDGKELKETLPDALKT
ECSKCTEKQKAGAEKVLRFVIENRPDDYKQVEAIYDPEGIYRQKFGAEAKEK
GIELPK
>TbraCSP14
DEGTYTTKFDNIDLDEILKNERIYEKYLLCLKDEGKCTPDGRDLKDSLSDALQ
SGCEKCSEKQKAGTQKVLRFVLEKKPNDYLALEKIYDPNRIYRIKYKDEAEK
LGLKFPNK
>TbraCSP15
DEGKYTTKYDNIDLDEILNNDRIYEKYFLCLKGEGKCTPDGRELKVALPDALK
TRCVKCSEKQRAGTEKVLRFILDKKPNDYLVLEKIYDPDRMYRVMYKEDAE
KLGLKFPTK
>TbraCSP16
MPLVFIGSIAAGEVYTSKYDNIDVDKILSNDRILSQYIKCLMDEGNCTNEGKEL
KKTLPDALATGCNKCSEKQRAQTEKVLRHLIKNRSRDWARLKGKYDPTGEY
SKKYETKVPASK
