# Supplementary material for: Identification and expression profile analysis of odorant binding protein and chemosensory protein genes in Bemisia tabaci MED by head transcriptome
Source: PLoS One. 2017 Feb 6;12(2):e0171739. doi: 10.1371/journal.pone.0171739 (PMC5293548; doi:10.1371/journal.pone.0171739)
Supplement: S2 File — (PDF) [file pone.0171739.s004.pdf]

S2 File. Sequences for MEME analysis

The MEME analysis of OBPs

>BtabOBP1
MMDLKAILLSLFVVTLSLVGITHAVTHAEMDQLAVACNGQKADIDVMKQWR
VPSSDSGKCLMKCLTDYYKMFDSEGKYSLKNTEDSLYKAWSEKPKEHMPIIA
QHCDNMMRSAPKENLGSCQMSYDTMKCINEKAWELGWFN
>BtabOBP4
MKSFIAVLAVLFVFACVQAQDAKQKVAAVAEKCKTEVHGGHDVLRIVGTSEL
PKTEEQRCFLECVYKNLQLIVDDKFAEPGAKRLMMMKYGSNPDQLKIAQTEI
ETCAKEVKPTAGMKCSLAHNIRQCFAKEGQKRNFYIKA
>BtabOBP6
MQGEVCFALLVVCLLHVTFIPDVETAVSEQMIKQMKDMHKKTCMARSGVKK
EGLAKFMEEGVNDDPAFKKYTLCMLKNMQGSKNGKIAIKDIENQAKAMLPP
PLRDAILDATSKCSNTGGDTPEDITYNFSKCSHKANVKSVMII
>BtabOBP8
MNNLMVFSILLYLQYLLAVKADQGNQGHQGQQGNQSEDQEKVKAIYQKCQ
QESMAEEKDLDNFKKMEMPSSEKGKCMMACLMREAKIIVNKRFSKDGAMA
LAQRYYSTQPQNMDKAKQVIVACDKQVEHERNECNIAGKLAECVVTEAQRV
GLTSVPKG
>ApisOBP1
MLNLKVMMFLCLSVIVVYCESDQVPINSSAAVESCLLETNMTRDEFEDMLTSP
NARELTILKSHAHKCMFGCVMRKNHIVNDGVVSKEVLSKYVLNFYGRPDYK
RRLIIKDVEHIVDVCAKKVADESETDECELAATLVTCIVLEANKAGLVDDPAR
QI
>ApisOBP2
MKVSAATAVLVALVATVQSSDPCNISTCYKSGTTKPPMAVTPTHLPVQSSSTQT
SHPQTTYAKDHVHGSTTTKSGVNATVTTASGASVNGTEPPAVVKSSAGVTGN
STTPKPTMTEGHVALKQKLNTIAVKCKDELHAPQEIMALVSNTVVPQNEQQR
CYLECVYKNLNLIKNNKFSVEDGKAMARIRFANQPEEHKKAVTIIETCEKEAV
IDPKTTEKCAAGRVIRNCFVKNGEKINFFPKA
>ApisOBP3
MISSTFYITLVFGIAMLISCGHGRFTTEQIDYYGKACNASEDDLVVVKSYKVPT
TETGKCLMKCMITKLGLLNDDGSYNKTGMEAGLKKYWSEWSTEKIESINNK
CYEEALLVSKEVVATCNYSYTVMACLNKQLDLDKST
>ApisOBP4
MRGNYSSMVFLLFAIGFQDIFCQKQEPSGKCRAPDKAPLNLEIIINTCQEEIKSA
LLQEALDILNDGNVEQNTPNYSSRSKREAEEDLTNEERRVAGCLLQCVYKKV
KAVDETGFPVVDGLMKLYNEGVQDRNYYIATLSAVRHCISIAQQLKQQQPSKS
FDDGQTCDLAYEMFECVSEKIEENCGVENKSNN
>ApisOBP5
MSANSATIKCIAVAAILLQISVIFADAGHHRRGKELLDTEDSDFFRCKQASRKS
CCGPENAMKRFGDKDKVAADECYAQVAEKFATVTATTPKQDLFSAEAVKITK
KKQFCLHECIGKKNNLLTEDGSLNKTFIADYAMKSVFKEQWQKQVGQKALD
KCLEETYIPWPAEDKENVCNPVYVQFQHCLWLQYESNCPANKIKITKKCEKT

RNRYRMQKSTSN
>ApisOBP6
MPNILPNLDSTWEKCFETFKQFKDKPETKEYKEMAHGKEPPCLFQCIFMQSGL
TTSDGKLNEDAITKKMSEGINNDEKWKSIWQNSLNKCFDDVKQEDKKQILIM
NTPAGRLMKCFLRDMYMSCPKNVWVESSECLNMKDLVQKCPEMPPPVFKSP
PKLI
>ApisOBP7
MVARKRMYNMLPTTVLFAIIAATVLKDCDAYLSEAAIKKTQQMLKTVCSKKH
SVEEDVFTNIKKGIFPEDNNNIKCYFACNFKTMQLINQKGVIDKKMFKDKMS
MMAPPNVYKILLPVIEQCTGKDKGEELCQSSYNVIKCAHSVDPKSLEFLPL
>ApisOBP8
MFALKVACLCLSVAVVFGENNQQNGPSDRSATIFQSCIAETKLSGDALKGFRS
MSIPKTQAEKCMMGCLMRKVNVINKGKFSVEEATKVAQKYYGTNEAMMKK
AKDLIDVCAKKAQSTTEECALAGIVTTCIVEEAQKAGLSGGPGSRSRRTVSPK
FRRDAM
>ApisOBP9
MIIKKTLLLSVFVLFGCLFSINKADDADAKDKELMSKLFTVVFKCFKDADWG
TCGEMITTKYDITQAKYKQCTCHMACAGEELGMINASGQPEPAKFLEYVNKI
NNPDIKSQLQLIYDKCQNVKGSEKCDLAEQFAICAFKESPALKERVSTLMEML
VKMKPKSK
>ApisOBP10
MEHLRSTNVVFAIVMALLVVQSSTRPQPDEMEEIKRTLYNACAGKFPITEEIKN
NAKNSIISDDPTFKCFLKCCFDEMSMIDEDGIIDGDSLKAMAPDHIKPILEQVIP
SCTKNVKQDGCEASFEFISCGIKLNPLIVALLPL
>ApisOBP11
MSSSTFYITLLFGIAMLISCGYGIFTTEQIDYYGKACNASEDDLIVLKSYKVPST
ETGKCLMKCMITKLGLLNDDGSYNKTGMEAGLKKYWSEWATEKIETINEKC
YEEGNTATLLYHVAIYFTCVSGDYSDVQLLIHCDGMFEQEVGSRQVNLKLLIM
LKIGLSEPKR
>ApisOBP12
DDLVVVKSYKVPTTETGKCLMKCMITKLGLLNDDGSYNKTGMEAGLKKYW
SEWSTEKIESINNKCYEEGDTSTLLYHVVIYFTCVKGGSSDVQLLVHCDGMFE
QAVGSRQVNL
>AlinOBP1
MNSLIPVLLVVCAAATRADEQTNAMVAKAFNKCREEFPISDDEIGGVREKTTI
PESHNAKCLMACMLREGKMLRDGKYEKENALIMADVLNKDDPASADKAKQ
LVETCAGKVGTDAGGDECEFAYKMAVCAAEEAKKLGVRPPDF
>AlinOBP2
MSLKIQFFVFAAICAACVCAYQEQLKQTIRDCQDGKEVTDDELEEFTKPLIPR
NREEKCIMACVMRTYNIISNGHYDPKIAFGILKGILKDHPEKLNKIKEVMDHC
GEDVPSHMDDECDLAGEIMQCEVKYQKAMGMA
>AlinOBP3
MDIRFGFIIACLAILSVANAISKEYSARMIAAKEKCQKEFNVTDSVVEDFMKR
NIKPESKSGKCMVHCIMEEMGMIDDHKINTEQVKLGNKEKWDDPALVELAN

QVADTCDQEVFTEGRCKCLVAVEYMMCLATHGDEVGLPHVDFEDSQDS
>AlinOBP4
MRIFVIFTAALTCVMAGELPEEMKEMAQGLHDSCVEETGVDNGLIAPCAKGN
FADDAKLRCYFKCVFGNLGVISDEGELDAEAFGSILPDSMQELLPTIKSCGGTT
GSDPCDLAMNFNKCLQKADPVNFLVI
>AlinOBP5
MVLKMNLLLVVLVMSQVFFSVTEAAMSQAQMKQAMKTVRNMCIPKSGVDK
EALAKMVNGEFDESDQKLKCYLGCVLGMMQAVKNNKINLTMVRNQITKML
APERGQRILAAFESCATVTGDDNCGLAFRFAKCIYDTDKEAFIVP
>AlinOBP6
MGFKFVKYRSYFFVLVIRIILCIQIKAKELTDEQKEQIFAEIKNCMESTKLTDEEF
ESIMAKKELPTSIEGKCFTKCLMEKMEYLEEGGKINVIAVQAGMEENMEKESE
ITKAKEVIQQCADSVPPEDSCEYAYGISQCMYNKMKEAGISGS
>AlinOBP7
MNRPLLLLTAVLTVGSGQQEDCKTAPAGWPRRPPQCCDLPFPLEGMKKEFGSC
IRQIGNRQSSAVPTAQAVRDARLCIEECVYKGLGFMDEHKLNKDQLLEQLKK
GIADKKDWTKPMEGAVKRCHETITKRETPQEAACQDSAHEFTHCAMRELFLN
CPASEWNNNDECNLVKSRMQACPNIPPPPPPPPQGFRGQGPPPQ
>AlinOBP8
MDTHFGLLIASLAILHTANAVINKDYLEKVVTAKDKCLKEFNVDDSVVEDFIV
KYNKPQSESGKCMVACFMEERGMMKDGKTITEQVMLDNQEKWIAATHVNM
GKEVIDTCDKEVPNEENDKCDLAVDYMMCLVKRGDEAGLPKMDVAQLKH
>AlinOBP9
MMELWKWRLALIIFGLVSCIQQTEGSQRTKQQPKSKTKESVVGATRPRDAKA
TECVNKVNANEEESASFFRKEIPETEAGKCLLACYLEGKGLIVGGKISSSGAAR
VAARAYPNNRVKTGNVKHILSHCGTIAGRESNNCEMAYKLADCTTTLSDKFR
L
>AlinOBP10
MFFNSVFLLVVCVSSYVTKGQELPPPGDVKNKTVVFKNSFLRSAKYCSSIYET
STLAIMALLMSEKSDDQNGKCFLNCMLQRYRLMSQDGSYNKDKFKPFLEYIP
DSKFLQSIRGNLKNCISEKDPDPCEKASKFIKCFYTRARNKGEIGASKEVIPAD
GF
>AlinOBP11
MKTFVGLIFAVALVEFASAISKEYHDKAIEAKNTCAKLHNVDDETIMTYWKN
HQLPEKEPETCIVICYLKEMKLVVDGKVDADAWKASNKEKWDDEKHVAAA
DEIVDKCSAEVPPTENECEWGLALTKCALKHGKEAGIPPPDMEHPKRR
>AlinOBP12
MTTKLRSIGLVFIVSISYAFAYQELLKETIKKCQNGRDVTDDEVEEFTKPLVPK
NEEERCLVACVFKEYKVIIDGHFDPVNALNVAKVVYKDYPDKVERIKDVLDH
CGEDIPTHNDNECDLAGDIMKCEVKYLNSVPKMTSLEFLAGSMAATAEP
>AlinOBP13
MNISTRMISLTMAYLAAALVSGHRALDGILPQANQDECREESNFRGELNDDV
GRNVTQELKCFAACSLMKLGIMNEKDGTVNMTRLDELIASHTPGKDAADVF
KTTVVEPCMKEVKKSTDYCEYSYQLIACGMSKVP

>AlinOBP14
MKPPGPPGSGTPPTAEERAARKIAHECADECLYKSSNLLTSAGELDKDAIKAL
VTKLYTGDWATAATTAIDKCLASAKGEVEATSKCKSGSFQLSRCFMRSMFLGC
PASSWTESTECAAAKARLTKCPNAMAPMPHKK
>AlinOBP15
MVDHWCASRIHKRYHELTHSRVVGGLRCCDEDEQTNAMVAKAFNKCREEFP
ISDDEIGGVREKTTIPESHNAKCLMACMLREGKMLRDGKYEKENALIMADVL
NKDDPASADKAKQLVETCAGKVGTDAGGDECEFAYKMAVCAAEEAKKLGV
RPPDF
>AlinOBP17
VKEIVQNVSKKCAAETKASPEQAKIILTQNIPKNDVERCYLQCVYSGVGVIKD
GKFSQEGGNKLVAMRFHDAKEKELAKQLINTCAKEIKAKDGEKCSLGKGIRQ
CFVAHGKEVNFFPHA
>AsutOBP1
MNSLIPVLLVVCAAATRADEQTNAMVAKAFNKCREEFPISDDEIGGVREKTTI
PESHNAKCLMACMLREGKMLRDGKYEKENALIMADVLNKDDPASADKAKQ
LVETCAGKVGTDAGGDECEFAYKMAVCAAEEAKKLGVRPPDF
>AsutOBP2
MSLKIQFFVFAAICAACVCAYQEQLKQTIRDCQDGKEVTDDELEEFTKPLIPK
NREEKCIMACVMRTYNIISNGHYDPKIAFGILKGILKDHPEKLNKIKEVMDHC
GEDVPSHMDDECDLAGEIMQCEVKYQKAMGMA
>AsutOBP3
MATNAKAVLFLALCGIVYVSAYQEVLKATISDCKGGKEVSQEELDEFIKPLIPQ
TREEKCLMACVFTAYNVIVEGHFDPKLAYGVAKNILHENPEKLKHIKETLDYC
GHEIPTKMDDECELASEVMACRNKYNKDHGYDQDP
>AsutOBP4
MRIFVIFTAALTCVMAGELPEEMKEMAQGLHDSCVEETGVDNGLIAPCAKGN
FADDAKLRCYFKCVFGNLGVISDEGELDAEAFGSILPDSMQELLPTIKSCGGTT
GSDPCDLAMNFNKCLQKADPVNFMVI
>AsutOBP5
MGHIPMSSDMTNILLLLVIGAGIAVNEGDASMEALMECKKDFKVSREQIMSG
DSSEEVKCFAECLMKKTGGMDEGGNFNTEKIKEEGRKHAKTDDQKRAHDAA
VDKCISETEAANPTGKCEKGFEFFKCVRGEMKSLM
>AsutOBP6
MGFKFVKYRSYFFVLVIHIILCIQIKAKELTDEQKEQIFAEIKNCMESTKLTDEE
FESIMAKKELPTSKEGKCFTKCLMEKMEYLEEGGKINVIAVQAGLEENMEKE
SEITKAKEIIQQCADTVPPEDSCEYAYGISQCMYTKMKEAGISGGP
>AsutOBP7
MNRPLLLLTAVLTVGSGQQEDCKTAPAGWPRRPPQCCDLPFPLEGMKKEFGSC
IRQIGNRQSSAVPTAQAVRDARLCIEECVYKGLGFMDEHKLNKDQLLEQLKK
GIADKKDWTKPMEGAVKKCHETITKRETPQEAACQDSAHEFTHCVMRELFL
NCPASEWNNNDECNLVKSRMQACPNIPPPPPPPPQGFRGQGPPPQ
>AsutOBP8
MKLALVTAFLSAIVLAEGNINKEYLDKLIAAKEKCVKEFSVDDSIVEDLYVRY

NKPPTESGKCMVACYMEERGMMKDGKTITEQVMLDNQEKWIAATHVNMGK
EVIDTCDKEVPNEENDKCDLAVDYMMCLVKRGDEAGLPKMDVAQLKH
>AsutOBP9
MMELWKWRLALIIFGLVSCIQQTEGSQRTKQQPKSKTKENVVGATRPRDAKA
TECVNQVKANEEESASFFRKEIPETEAGKCLLACYLEGKGLIVGGKISSSGAA
RLAARAYPNNRVKTGNVKHILSHCGTIAGRESNNCEMAYKLADCTTTLSDKF
KL
>AsutOBP10
MFFNSVFLLVVCVSSYVTKGQELPPPGDVKNKTVVFKNSFLRSAKYCSSIYET
STLAIMALLMSEKSDDQNGKCFLNCMLQRYRLMSQDGSYNKDKFKPFLEYIP
DSKFLQSIRGNLKNCISEKDPDPCEKASKFVKCFYTRARNKGEIGASKEVIPAD
GF
>AsutOBP11
MKTFVGLIFAVALVEFASAVSKEYHDKAIAAKNTCAKLHNVDDETIMKFWKA
HQLPEKEPETCIIICYMKEMKLVVDGKVDADAWKASNKEKWDDEKHVAAAD
EIVDKCSAEVPPTENECEWGLALTKCALKHGKEAGIPPPDMEHPKRR
>AsutOBP12
MFQAFVYQELLKETIKKCQNGRDVTDDEVEEFTKPLVPKNEEERCLVACVFK
EYKVIIDGHFDPVNALNVAKVVYKDYPDKVERIKDVLDHCGEDIPSHNDNEC
DLAGDIMKCEVKYLNSIPKMTSLEFLAGSMAATAEP
>AsutOBP31
MGRLTKTQNADLLIHPAGKIRVGVLPELFVGGIQDVIPCHDEARLKKPSGRHG
GGGSPPSAEDMAAHLRAHECADECVLKSQNLLTTDGELNKDAIKAQVVKSFT
GDWAKLASDTADKCLASAKGEVTATATCKSGAGQFIYCFRRNLFLQCPSSSW
TETTDCAAAKARITKCPNAKIPMGHHRH
>AsutOBP32
MDVMILPVFLIVFIAATASPAVLSAECPQRFPKEMRPLSSCCTVEMNPNSSYNY
TEGDPIINKCFGDFNSSATRPTGPPSGYDCELECLMIEFGYMGKDKTINKDKIV
KSIEDEYSADFQEAGHKAIEICMGRKYHTSCPSGIDGMIECFAVQMMLNCPAK
HWSGGEDCKETKQLIDKCGEVLSIFADYDTD
>AglyOBP2
mkvsaatavlvalvatvqssdpcnistcyksgttkppttvtptrlpvqssstptshqqttyakdhvhsstatksgvnttattt
sgasvngterttvvksssgvagnvttpkptmtdghlalkqklntiavkckdelhapqeimalvsntvvpqneqqrcyle
cvyknlnliknnkfsvddgkamakirfanqpeehkkavtiietcekeaiidpkttekcaagrvirncfvkngekinffp
ka
>AglyOBP3
misstfytslmfgivmliscsfgrftteqidhygkacnateddlvvvksykvptsdtgkclmkcmisklgllnddgsyn
ktgmeaglkkywsewstdtiesinnkcyeeallvskdiiatcnyayvvmaclnkqldldkst
>AglyOBP5
mkrfgdkdkvaadecyaqvaekfatvtattpkqdlfsgeavkitkkkqfclhecigkknklltedgslnktfiadyamk
svfkeqwqkqigqkaldkcleetyipwpaeetenkcnpvyvqfqhclwleyesncpdnkikltkkcektrnryrmq
kstsn
>AglyOBP6
mqkvvflcifaiicqtvftvgfertwilrqkrvtnddecrtlipssekklptccqmpnilpgldnawevcfekfkqfkdk

hatkeykemahgneppclfqcvfmqsglttsdgkvnedavikkmaegmdndekwksiwrntfnkclndvkqed
keqikmtntptgrlmkcflrdlymnrpknvwvessecsnlkdlvekcpkmpppvfksppkli
>AglyOBP7
mvarkrmymlpatvllavvaatilkdsdaylseeaikktqkmlknvcskkhsveeevftdikkgifpennnnikcyfa
cnfrtmqmvnqkgildkkmfkdkmtmlappnvlaillppieqcigndkdteicrssynfikcahrvdpksleflpl
>AglyOBP8
mfafkvaclclsvavvfgennqqnsndrsasifqscisetklsgdalkgfrsmsipktqaekcmmgclmrkvnvinn
gkfsveeatkvaqkyygtnetmmkkakdlidvcakkaqstteecalagivttciveeaqkagltggpgsrskrtvspkf
rhsiv
>AglyOBP9
miikktllvsgfvlfgcmfsinkaaddadakdkelmsklitvafkcfkdadwgtcgemittkyditqakykqctchma
cagedlglinsngqpepakfleyvkrinnsviksqlqhiydkcqnvkgtekcdlaeqfaicafkespemkervtklie
mlvkmkpksk
>AglyOBP10
mehlrgtnvvfaivmallvvqsstrpqpdelddikktlynacsekfplteeiknnvknsividdqnfkcflrccfdemsl
idedgiidgeslaamavdkikpvaekivhdclpagkqekqdgceasfkffscgiklnpltiellplq
>AglyOBP11
misstfytsllfgiamliscsfgrftteqidhygkacnateddlvivksykvptsdtgkclmkcmisklgllnddgsynkt
gmeaglkkywsewstdtiesinnkcyeeggticfilnrfvt
>AgosOBP2
MKVSAATAVLVALVATVQSSDPCNISTCYKSGTTKPPTTVTPTRLPVQSSSTPTS
HQQTTYAKDHVHSSTATKSGVNTTATTTSGASVNGTERTTVVKSSSGVAGNV
TTPKPTMTDGHVALKQKLNTIAVKCKDELHAPQEIMALVSNTVVPQNEQQRC
YLECVYKNLNLIKNNKFSVDDGKAMAKIRFANQPEEHKKAVTIIETCEKEAIID
PKTTEKCAAGRVIRNCFVKNGEKINFFPKA
>AgosOBP3
MISSTFYTSLMFGIAMLISCSFGRFTTEQIDHYGKACNATEDDLVIVKSYKVPT
SDTGKCLMKCMISKLGLLNDDGSYNKTGMEAGLKKYWSEWSTDTIESINNK
CYEEALLVSKDIIATCNYAYVVMACLNKQLKLDNST
>AgosOBP4
MRGNYSLVVFLLFGFGLLEIYCQKQELSGKCRAPDKAPLNLEIIINICQEEIKSA
LLQEALDILNDGTLEQNTPSYSRSKRDADEDLSNEERRVAGCLLQCVYKKVK
AVDETGFPVVDGLMKLYNEGVQDRNYYMATLSAVRHCISIAQQLKQQQPSKS
FDDGQTCDLAYEMFECVSEKIEENCGVENKLNNLSQRQV
>AgosOBP5
MKMSANGATMKCVAVAVVLFQMSVIFAEAGHQRRGKELLDTEDSDFFRCKQ
ASRKSCCGPENAMKRFGDKDKVAADECYAQVAEKFATVTATTPKQDLFSGEA
VKITKKKQFCLHECIGKKNKLLTEDGSLNKTFIADYAMKSVFKEQWQKQIGQ
KALDKCLEETYIPWPAEETENKCNPVYVQFQHCLWLEYESNCPDNKIKLTKK
CEKTRNRYRMQKSPSNQ
>AgosOBP6
MQKVVFLCIFAIICQTVFTVGFERTWILRQKRMTNDNECRALFPSPEKKLPTCC
QMPNILPGLDNAWEVCFEKFKQFKDKHATKEYKEMVHENEPPCLFQCVFMQ
SGLTTSDGKVNEDAVIKKMAEGMDNDEKWKSIWRNTFNKCLNDVKQEDKE

QIKVMNTPTGRLMKCFLRDLYMNCPKNVWVENSECSNLKDLVEKCPKLPPP
VFQSPPKLI
>AgosOBP7
MNMLPATVLLAVVAATILKDSDAYLSEEAIKKTQKMLKNVCSKKHSVEEEVF
TDIKKGIFPENNNNIKCYFACNFKTMQMVNQKGILDKKMFKDKMTMLAPPN
VLAILLPPIEQCIGNDKDTEICQSSYNFIKCAHRVDPKSLEFLPL
>AgosOBP8
MFAFKVACLCLSVAVVFGENNQQNSNDRSASIFQSCISETKLSGDALKGFRSM
SIPKTQAEKCMMGCLMRKVNVINKGKFSVEEATKVAQKYYGTNESMMKKA
KDLIDVCAKKAQSTTEECALAGIVTTCIVEEAQKAGLTGGPGSRSKRTVSPKF
RHSIV
>AgosOBP9
MIIKKTLLVSGFVLFGCMFSINKAADDADTADKELMSKLITVAFKCFKDADW
GTCGEMITTKYDITQAKYKQCTCHMACAGEDLGLINSNGQPEPAKFLEYVKR
INNSVIKSQLQHIYDKCQNVKGTEKCDLAEQFAICAFKESPEMKERVTKLIEML
VKMKPKSK
>AlucOBP1
MKSFVGLIFAVALVEFASAITKEYHDRAVAAKDACLKKHPSIKESDVQEFLKK
HKLPETDDGKCMIACYMEEMNLMADGKINVEEAKKTNSDKYDGEPDNKEL
ADKLIDHCSSQVSPDGMSKCEYAYQISKCGLEYGMKNGLTPPKMYEEQRR
>AlucOBP2
MTYHVFFRKFDLPRISRRVRQCYYHSVPRSLSGSSRRMLEETSQHHPKRRSRV
SEKHKLPETDDGECMIACYMEEKNLMADGKINVKEANQTNSDKYDGEPDNK
QLAEKLIDHCSSQVSPDGMSKCEYAYQFSKCGLEYGMKNGLTPPKMYEEQRR
>AlucOBP3
MKTFVGLIFAVALVEFASAVSKEYHDKAIAAKNTCAKLHNVDDETIMKFWKA
HQLPEKEPETCIIICYMKEMKLVVDGKVDADAWKASNKEKWDDEKHVAAAD
EIVDKCSAEVPPTENECEWGLALTKCALKHGKEAGIPPPDMEHPKRR
>AlucOBP4
MDTHFGLLIASLAILHTANAVINKDYLEKVVTAKDKCLKEFNVDDSVVEDFIV
RYNKPQSESGKCMVACYMEERGMMKDGKTITEQVMLDNQEKWIAATHVNM
GKEVIDTCDKEVPNEKNDKCDLAVDYMMCLVKRGDEAGLPKMDVAQLKH
>AlucOBP5
MNSIIVLCLVASAVTLSQGNPTTPNPSTSHVSSSAGITVSGVSKSPEEIKLKIKEQ
VATLTGACKTQTKLTGEQAKIVASQAIPKTEAEKCFLECIYQGLQLTKDGKFNE
PAARAWAQKRFGNAPEDLQKANTMIDICVKEVVVKDENEKCALGRLIRECFV
KNGAKINFFPKP
>AlucOBP6
MYDRFKLFALLALVVSCKSAPPEEPAECKLPESDSAELVKCCKLNVVLDEMA
DSVGECMKLVKGKPEKGPPVPEGFDCMDTCVFSKLGFAANNKLDAEKLTKK
FSELFKGDWSALSDSTLKKCLPMAEGAKGSCASGADVFKFCIVRELYMNCPA
SSWTKSDLCKANVERLEKCPHSMPFLPGTGIKKN
>AlucOBP7
MNPLILILLVVFAAATRGEEQANALVAKAFNKCFGEFPLGDDEMKEVKDKST

VPSSHNAKCLMACMLKEGRILRGGKYELENAILMADVLNKNDHAATDKAK
QLIETCAAQVGTDASADECEFAYKMALCASDEAKKLGVRPPDF
>AlucOBP8
MVLKMKQILVVFVALQVLISTTEAVMTQAQMKQAMKTVRNMCIPKSGVDKE
ALAKMVEGEFDESDQKLKCYLGCVLGMMQAVKNNKINLTMVKNQISKMLA
PEQGQRILAAFEGCATVTGDDNCDLAFKFAKCIYDTDKELLFQAFIVP
>AlucOBP9
MKSFVGLIFAVALVEFASAITKEYHDRAVAAKDACLKKHPSIKESDVQEFLKK
HKLPETDDGKCMIACYMEEMNLMADGKINVEEAKKTNSDKYDGEPDNKEL
ADKLIDHCSSQVSPDGMSKCEYAYQISKCGLEYGMKNGLTPPKMYEEQRR
>AlucOBP10
MTYHVFFRKFDLPRISRRVRQCYYHSVPRSLSGSSRRMLEETSQHHPKRRSRV
SEKHKLPETDDGECMIACYMEEKNLMADGKINVKEANQTNSDKYDGEPDNK
QLAEKLIDHCSSQVSPDGMSKCEYAYQFSKCGLEYGMKNGLTPPKMYEEQRR
>AlucOBP12
MTCSHFIALLSVVALSLSSGEINEECKDIENLKTQLENFYGCCDFESMIERVVR
TEEEVETDRFCREERKKINSTDGKVPLASEGHDCFMECVLKRMGAMGQDFK
FIREKLDDFFLRGYPEEVKQAGKLAFDKCLSKNFSKKYCASGINGLMMCLPE
ELVMNCPANIWSSHESCPIAKEAIKKCPSYRVMIEQE
>AlucOBP13
MKHSSCVVPVALTIFVVAIVSGFKELDDVLPKPKQDECRKESNFQAELPSDINQ
NITQELKCFAACSLVKLGLMNEKDGTINMAQLEDLIAKHTGGKDAADMFKH
TVVEPCMKEVNKTTDYCEYSFQLVKCGMSKVKPPSTGTEG
>AlucOBP14
MALNAKAVLLLGVCGLVYVSAYQEVLKATLKDCKGGKEITQEEVDEFMKPLI
PKNEEERCLMACVFRAYNVIVDGHFDPKLAYGVAKNILHENPEKLKHIKETLD
YCGHEIPTKMDNECDLAGEVMSCRNKYNIDHGYDQDP
>AlucOBP15
MMRPTAYYLFASYAALLVCVHFASVSAITPELDKRAKAAVAKCADVPRTDEA
KKEDCHAGCFMSAMGYMTNGEINVKNMEEANKQKWDDQEIIKKGIQVDTT
CAKQVGDTKGKSECTIGYEFSTCKKELVKKVGLPPPTPLKE
>AlucOBP16
MKRLVFVLFTLCSLQWVSGITDELKQKAQAARLTCKQQVGLSDKEFNDWVK
GIALPTTDGGTCCEVCACWMRELGYLTGGRVNLENMKAVNAQKWNNLAYV
ELGNKIDALCSDRVLQTGRKECEIAVDFRKCKTELIQQFGGPPKPGST
>AlucOBP17
MRILVLFTAALTCVLAGELPEEMREMAQGLHDSCVGETGVDNGLIAPCAKGS
FADDPKLKCYFKCVFGNLGVISDDGELDAEAFASILPDNMQALLPTIRGCGST
TGADPCDLAMNFNKCLQKADPVNFMVI
>AlucOBP18
MHAAIVLIGSALLVAYVSGAPSANVKEIVQNVSKKCAAETKASPDQAKIVLSK
NIPKDDAERCFLQCVYTGVGVIKDGKFSEEGGKKLVALRFHDAKEKELANKL
IATCAKEIKAKDGEKCSLGRAVRECFVNHGKQVNFFPSA
>AlucOBP19

MNSRFGIVFASLALLHITNAGNIKEGYVAKIAEIKDKCLKEHNVDHSVVEDLL
KKSIKPEVKAAQCMVACFFEENGMMKDGKIVSEMVKSNNAHQYEDPADVEK
ANEASDMCDGEVSTDGKDKCLLAADYALCWVKRTEEAGLPQIDFANSS
>AlucOBP20
MYTFKTFFVLTLASYVIAAPPADEPAECKPMKEKEEEISKCCKLEPVTVKEQA
AFVDCMKLVKDTDKKGPPKPEGFECLDDCILSKTGSLGSDKKIDPAKINAAAK
TTYTGDWAEPGAKMVEKCLAQVAENKDKTVCSTSGADVYTKCIFRESYINCP
EKSWTNSDACKANKERVIKCPKTLPYNAEQHKAETR
>AlucOBP21
MKFFVVSAALVLLVAAAVKANEKKANEKVTEIFNKCKETWPVTDEEIEQVKQ
KQSIPDSKNVKCILACMLKEAKILRDGEYNKDNAELMADVLYKDEPEHAEKS
KQIIEMCSSELGTKTEGDDCEYAYKMSVCASKHAKELGVKTPEF
>AlucOBP22
MSLKIHFFVFAAIGAACVCAYQDQLKQTIKDCQGGKEVTDEELEEFTKPLIPK
NEEERCIMACVMRTYNIINNGHYDPKIAFGIIKGILKDHPEKLDRIKEVMDHCG
EDVPQHMDNECDLAGEIMQCEVKYQKAMGLN
>AlucOBP24
MSTKLRSVGMILAIAITHVCAYQEQLKETIKQCQDGREVTDDEVEEFTKPLVP
KNQEERCLVACVFKEYKVIIDGHFDPVNALNVAKMVYKDYPEKWKRIKDVID
HCGEDIPTHNDNECDLAGDIMNCEVKYLNSMPKGVSLELLAGSIAATAEP
>AlucOBP26
MNPTVAIIFTLLVAYVKANTKELSPSEALKQKVKVQCQQEVKATPEQLKIYDN
FKDVPKDDVENCLMECMYTKTGGIGADGKYSVEGFKKLVDMKYKGEENTK
ARKIAADCEAKAAPKEGEKCSMGRAIRECLAAATKENEFFTI
>AlucOBP27
MARKFIKSCYTLVALLVFVGSIHVEAKELTEEQRTQLFEDLKQCKNSTDLSDD
EFETIIAKKELPTSEAGKCFTKCLMEKLDIIEDAEGGKKKISVITMQASLEENM
EKEDDIAKGKDIIQKCGDTVEPEDSCAYAYNISKCIYDRMKEAGISQ
>AlucOBP28
MIIEIICVLTVGISPHFIEGQELPPPGGVGNKTAVFKESFIRTAKYCSSIHETSTVA
VLAILMSEESDDQNGKCFLNCMLQRYQLMSKQGAYNKDKFKPFLDYIPESRF
LQSIKGNLKTCITERDPAPCEKAYKFIKCFYTRARNKDEFGKIQRK
>AlucOBP29
MNRPLLLLTAVLAVGSGQQEDCKTAPAGWPRRPPQCCDLPFPLEGMKKEFGS
CIRQIGNRQSSAVPTAQAVRDARLCIEECVYKGLGFMEEHNLNKDQILQQLTK
GVADKKDWTKPMEDAVKSCHETITKRETPQEGTCKDSAHEFTHCVMRQLFLS
CPASEWNNNDECNLVKSRMQACPNIPPPPPPPPQGFRGQGPPPPQ
>AlucOBP30
MNAHIVLCLVASVFALSQGTPTTPTPATSRRVTVAPEDLEQAKSLRKFCTAKTG
FTGITTTETSKGKDQARTATTPRPKTQLEKCYLECLYTGLQLTKDGKFNEPGA
RALANKRYKNAPEELRKVNSIIDFCITEVVVRDIEEMCALGRLIKECFSKYGA
KNFPEL
>AlucOBP31
MFTSATFTVFLFAVTLTRGQIDEDPECRPSGPPGKEPECCTIPMKLFGDEVQEA

VVKNCFDEAGMKRPSGPHGGGSPPTAEEMAAHISAHECADECVFKSGNFIKS
DGGLDEDAIKAVIAKLFTGDWAPIATAAVNKCLASAKSGVSASAKCKSGAYQ
LSKCFQRELFLGCPASLWTESTDCSAIKARITKCPNAKVPIGHHHKH
>AlucOBP32
MSGRHSLILVLLAAVTSAEVLTDGDCPKTMPKEMKPLYKCCVVEMDSNKTIS
DDQKAAVDSCVNTSKSDSDANKHDCMIECIFIKLGYMGEDKTINVDYVLKEM
NSLLPEDFHEQTSKSLATCMGKKFSSTECPSEIDGVMACFSTMVLMNCPAKH
WTDDEECKATRKFFQKCGDSIGYRYD
>AlucOBP33
MHPWKTTCLIGMTAALMVVTAFAGLPFQNEMAVMQCKVKFDVTAEDIQLLK
DSKLPSSHSGKCMMACILKKMKVMTKRGQFDLRNVQKWLRNKYQGDQAN
LAKGNYVAEACANTLPTLGIQDECEMAAEIMTCVRTKSKLVKKTLNGELPKE
VSP
>AlucOBP34
MEHWKWRLALLIFGMVTCVPQLEGAQKSKQPSKAKTKESQVVAARPKDAR
AAACVTQIGPDEEEEASFYRKEIPETDKGKCLLACYLESKGVLSGGKFSSSGA
AKIAARAYPNNAAKTGNVKHILSHCGTIAARETEQCQLAYRLAECTTTLADK
FKL
>AlucOBP35
MINVVFVLLIGTGIVSGGFMEALIECKQQHHVSKEEAMTGESEEVKCFSECVL
KKSGMMSDNNEFDEEKIQAEGARMIKNDEQKNREFEGAAKACIEKVNGENP
SEKCAKGHALFKCMKEAMPMSKMRG
>AlucOBP36
MKTFVGLIFAVALVEFASAVSKEYHDKAIAAKNTCAKLHNVDDETIMKFWKA
HQLPEKEPETCIIICYMKEMKLVVDGKVDADAWKASNKEKWDDEKHVAAAD
EIVDKCSAEVPPTENECEWGLALTKCALKHGKEAGIPPPDMEHPKRR
>AlucOBP37
MDTHFGLLIASLAILHTANAVINKDYLEKVVTAKDKCLKEFNVDDSVVEDFIV
RYNKPQSESGKCMVACYMEERGMMKDGKTITEQVMLDNQEKWIAATHVNM
GKEVIDTCDKEVPNEKNDKCDLAVDYMMCLVKRGDEAGLPKMDVAQLKH
>AlucOBP38
MGFKFVKYRSYFFVLVIHIILCIQIKAKELTDEQKEQIFAEIKNCMESTKLTDEE
FESIMAKKELPTSKEGKCFTKCLMEKMEYLEEGGKINVIAVQAGLEENMEKE
SEITKAKEIIQQCADTVPPEDSCEYAYGISQCMYTKMKEAGISGGP
>LstrOBP1
MNVKMSALLKVGLVFLVYLCSYSGAIPALTEAQIEQVGKAMANMCISSSGVQ
RSLITKAMKGEIEDDRKLKCFFGCIMEAVQVTKNGRMQPEVLKRRANAMLPK
TMREMILPTIDSCSHIENEDKCELAYSIVKCHFSVNGKNPFFFNF
>LstrOBP3
MERTRLFIILAFMPFLPSALGANFAMMQQSLQGTNIPMIQSIAGELKFCMDVN
SEQNSDGLDDYLPLLFNEELPTTLGQKCFLTCLFNRFGLLKDGFLDTQTAKTL
VETFYKDKHDEKTMANIAINVCHVSAVPDVLNPCEIGFSLKSCFVDSNKKGKE
LRHKN
>LstrOBP4

MKSLIVCVVVSCLLVANTKADEATSSKPSSSPNAADALIASTTLSPASNETDAA
RAAIKEQLAKLTESCKTSSQANSDEAKIIGTESVPKTEGEKCFLQCVYTGLGIV
KNEQFSVEGAKLLAQKRFGSFPEELEKANQLIETCSKEAVKKDSKDKCPMGF
LIRQCFVKNGQKINFFPKA
>LstrOBP5
MKCQVLLALFVVAAVCEVCYAGLTPEKLKELKPLIDTCIKQSKAEEETIGKLQ
NGHEIPSSQTGKCFIACMAEHMKLMKDGKFEPAMTMEFIDKMVQDKDKAAE
IKKAVDDCFKSVPDTGDKCEMAAGLATCMKEHHAELAGMN
>LstrOBP6
MLYVLYFVIVTSALSAVITQIMAADSSDMLTVFNKCRDKTSATEDDIKTFRAQ
QIPSTTTGKCMLACMFNHSGLMKDGKYDSEGALKLVGQVFADNPIKLGKAR
QLINGCTDEVKKENDKCEIASKIADCTVKMSSQVGLS
>LstrOBP7
MKASAALTLVFLALAVFHCSEAKIDKAKKEAAIKKCQAETSASDEDVKKVRK
EHVVPESEEGKCFIACGFNTYDMLKDNRINLEGVNAFFEKLYDEQEKRDIAIK
AAASCAATESISGLNECHVAAKYFACLQRHPDFVKMKEDFDV
>LstrOBP8
MITSALTPSAVLAACLLLATAYAYDFSDPYFNEHLQSAMEEIMEEEMLSIGRVQ
RDADQGQEVADEYFKCKHRNLKTCCGKINLMKNYGDKGKIYGKQCYEEVV
SAFKSNSSSTADDDDSMMDMFSCEKVKMIKLKHICVHECIGKKTKILKEDGSL
NAEEIKQYAREYMFNEEWSKELGEKALDKCLTQTYNSVTKMLDEYEIKCNPT
SVQFHHCLWKEIEMTCPESKVDLKAKCVRLRERLRKQQAAGM
>LstrOBP9
MSRLHKFAISGMAVLAGAMLIAAEDTTIKIKNPSPHKQQQVYCQAPPTAPERL
ERIIEQCQDDIKTALLQEALNVLTDTSPRDLVKKTRSKREVFSGEEKRIAGCLL
QCVYRKVKAVDDQGMPTVPGLVRLYSEGVQDRNYYVATVQAVQQCVSASQ
HFRYYNPQVLKEDGYTCDLAYDMFNCVSDKIEAFCGRTP
>MperOBP3
rfsteqidyygkacnaseddlvvvksykvpttetgkclmkcmitklgllnddgsynktgmeaglkkywsewstekie
ainnkcyeeallvskeviatcnysytvmaclnkqldldkst
>MperOBP6
pnilpgldstwekcyekfiqfkdkpetkeykemshgkeppclfqcifmesglttndgklnedaitkkmteginndek
wkstwkksldkcfddvkqedkkqilimntpagrlmkcflrdiymncpenvwvesseclnvknlvqkcpempppv
fqsapkli
>MperOBP7
ylseaaikktqqmlktvcskkhsveedvftdikkgifpennnnikcyfacnfktmqminqkgtldkklfkdkmsm
mappniynillpaieqcigidkgeelcqssynfikcahrvdpksleylpl
>MperOBP8
ennqqnssdrsatifqsciaetklsgdalkgfrsmsipktqaekcmmgclmrkvnvinkgkfsveeatkvaqkyygt
netmmkkakdlidvcakkaqstteecalagivttciveeaqkaglaggpgsrsrrtvspkfrrnsm
>MperOBP10
strpqpdeleeikktlynacagkfpiteemkkdilnsnmvddqnfkcflrccfdemsmidedgiidgeslismatdnl
kpviqqivqscvkdikqdgceaafnfiscglklnpmtiqllpl
>NlugOBP1

MKSFIVCIAVSYLLVANIKADEATSSSDAESLITSTTLSPASNESDAARSAIKEQL
AKLTESCKTSSQANSDDAKIIGTESVPKTEGEKCFLQCVYTGFGIVKNDQFSVE
GARLLAQKRFGAFPEELEKANQLIETCSKEAVKKDSKDKCPMGFLIRQCFVK
NGQKINFFPKA
>NlugOBP2
MKCQIVLAALALATICEVSYAGLTPDKLKELKPLIDTCIKQSKVEEDTLGKLHN
GHEIPSSQSGKCFIACMAEHMKLMKDGKFEPEMTMEFIDKMVQDKDKAAEI
KKSLGECIKSVPEGDKCEMAAGLATCMKDHHAELAGMN
>NlugOBP3
MKASAAITLVFLSLAVFHCSEAKLDKAKKEAAIKKCQAETQATDEDVMKVRK
EHIVPDSEEGKCFIACGFNSYDMLKDNRINLEGVNAFFEKLYDEQDKRDIAIK
AAASCAATETVSGLNECHYAAKYFACMQRHPDFAKMKDDFDI
>NlugOBP4
MERTSVLIVFTFIPFLSSVLGANFLMMQQSMQGTQMPMIQSIASELKFCMDVN
AEQNSDGLNDYLPLLFNEELPSTLGQKCFLTCLFNRFGLLKDGFLDTKTAKNL
VETFYADKHDEKTMANIAINVCHVAAVPDALNPCEIGFSLKSCFVDSNKKGKE
LRGKN
>NlugOBP7
MLLEVCRFSVFLIALFATVNGRFTEEEKQLMNQVHSQCISETGTSEDLVTKATT
GDFADDDNLKCYVKCIWSTLTVMDDEGNFDVGVLEVMLPADMKDTVMKA
MNACTGVGGATPCEKAFAMTKCLYKEAPSDFFLP
>NlugOBP8
MVTSALMQTATAACLLLVTAYAYDFSDPYFNEHLQSAMEEIMEEEMLSIGRVQ
RDADQGQEVADEYFKCKHRNLKTCCGKINLMKNYGDKGKIYGKQCYEEVV
SAFKTNSSSTADDDDSMMDMFSCEKVKMIKLKHICVHECIGKKTKILKEDGT
LNPEEIKQYAREYMFNEEWSKELGEKALDKCLSQTYNSVTKMLDEYEIKCNP
SSVQFHHCLWKEIELTCPESKVDLKAKCVRLRERLRKQQAAGM
>PsolOBP1
MKLLPVLLLLVGLLAAASSHHLHEALSDEDKQRHHQESEECIAESKLDPQILA
DMKAGKKPDPIPRELHCYAKCILKKEGVMKEDGSINEDRPGRSDAAKECEDK
AKPASLAEADQCDTAGKIMGCYAKNHLIPKF
>PsolOBP2
MQKFICVAVAILVISSVAAAFAPNDVHEAAGKCRLENGIQSDAELNNVNDRKV
KCYFGCFMREMGMIVNGKVNPDKEIELIKTITPEQYNEEIKAKVYECANITSVI
TDVCELGMTSYLCVFGKV
>PsolOBP3
MKYLIAICALCFVSCISALTDEQKAKLKSYKEGCIYESGLNPAVIEQIKKGGSVP
FDDKLNCFSACLLKRLAIMKPDGSIDEAVARAKIPKEVPQNKANQVINICKSQ
VGRTQCETGGKVLGCLLKTKAVQFL
>PsolOBP4
MYTNIIIFVIAISYGPLCQSQDFLEKEAQVAGSEYMQGIVRYCTALHDTNTEDI
VKTYNYEVSDNKAKCAVMCILQSVQIMDQNGQFREDELQSFLEKVPEPEKRE
EIVTTLNDCVDTGGNNPCEKAFVFTKCLNKLRDMLPQSMVPAL
>PsolOBP5

MYFSVVFLFVLNTLIVQGTSTGSLSFYTDQEHQALQLCSQEYQTVLDTPYFVA
NARIPDESDHNMKCLFYCVPQKIGVTDDKGVINSVQIRDMLKTKYPSMSNTA
VKILLKKCGKERKTMDKCDKWYEVSKCTWKLYLEYQEKIEAKRQKKTTSPR
PSTTHRAHHHLKKENTPIPEIINH
>PsolOBP6
MLALVAFLFVASCYAAPQEMGSHVYEAEGDCFDAFPSITRDYLEGLKKVKKL
PENPTRDFKCFITCVGKKIEAMSANGEFDFDTLKEITVLITGGEATHDDAHKM
VSNCFDVEHDDDCEKAWKYIECKIEELKPYKFSKGFCPTCHE
>PsolOBP7
MYLSTVSVIFSCIFALTQAAYDQKQILELTTKCKATPEDTRTALNYTVPETPTG
KCLMLCMIKELNMLDSNDKYDEVLGLAALRKYWTEIKDAKLLDVNTNCVAY
AKTLPPDTPSCEYGYKIMKCINSEFIKGNLFEESFKKPIKIE
>PsolOBP8
MNKFVAVLFASCLVALVSADEAEINKQRAAAGEACKKEQNVGDDVAAILTSK
QIPSTEAQQCFLECFYGKLGLVKDGKLNAQGAQAIAKAKFGDDKDKLALAD
NIFKKCEEEVAKVASEKCGLGKAIRTCFVNNGDQIQIFPKSQ
>PsolOBP9
MKYLASFVFLLGVIVVVKADGAKSSTVPPTTVKSATTVDAKNNKTANLTFSK
NLKANDSVHNRFNAVATECKKELNANQEIMALLSSGALPTNEKQRCFLECVY
NKVGLIKDGKVNEEGAMSLAKAKFGENKDLMTKAEALFKKCKTEAVVEKDS
KEKCALGRLIRTCIVNHGNNLPIFSKAS
>PsolOBP10
MKDLSFILFITFCGLTYAAIEKEQAVTDCVTELSIDEEIRAHFKNHGEIPDETDK
NAKCLFHCVSKKMDFTDEDGSIHKEKVIDYIMQKYPDLNKAKIEPVVLLCGE
RSETDPCEKWYEFTKCQLKAYLEYKKTQ
>PsolOBP11
MAHIFKIVTVYILGFMYSVNGESKLMSIMIENLMDCMAQAPENFSLDVCQEM
LRDGNDNSLVKYDSCKCLGACTSKKLGIMDANGIGVKSKVEEYIAQLDNEV
WRNEAIKILKKCADTPGKNCDLSYNFMVCTMENSSLVRDFVKAMTSGDKSK
EEN
>PsolOBP12
MLSFALIVFAFFQLGFAVDKKEIMASCMQQYSVGEEVFKNFLQNGEVPDESNK
NQKCLFLCVISKLGMSDESGNMDMPKVKEMMVAKYDKMTPENVIALVEKCA
VRSEADPCDKIYQFHKCHMKPYLEVVNG
>PsolOBP13
MNSLGICVIFSCCLFVLTQAAWEKAHIREAISKCNATEKDADVLLTKFSAPDTP
SGKCLMLCVLKFYNMIDSLGKYDEAAGLARLRERWTEIDDAEVVAANSNCV
AFGKTIPDASACEYGYQIMKCLIQEVTNGKFYEKSMKKLAEKKEATTSN
>PsolOBP14
MSGMFKNCLLILSFVCLTCYVNCEGELPTKGISQRCKTPAIAPQKLERIIGQCQ
EEIKSLLLQEALDVIGIENQDGSLTTPIRNKRQTKEQTASFTNEERRVAGCLLQC
VYKKVKAVDESGFPQVDGLVRLYSEGVQDRNYYLAAYTAVQQCIGIAEAVKQ
QQPSQKFDGGQICDLAYEMFDCVSDKIDQFCGLTPERV
>PsolOBP15

MFKLTSLILLFCVSTHVFADDIPEELKEMIKNLHNQCVGETGVSEAAIADANN
GKFNGEESLKCYMKCLMASVGIIDDDGVFDGEAFVDLLPNSMKSHGEKLVK
ACKPEGSNACDVAYNLNLCNYNLDPSKYTLY
>SfurOBP1
MNSLIVCVVVSCLLVANIKADEATSSSSPDADSLITSTTLSPTSNETDAARASIK
EQLAKLTESCKTSSQANSDDAKIIETESVPKTEGEKCFLQCVYGGLGIVKHDQ
FSVEGAKLLAQKRFGSFPEELEKANQLIETCSKEALKKDSKDKCPMGFLIRQC
FVKNGQKINFFPKA
>SfurOBP2
MKCQVFLASFVLVAVFELGYAGLTPEKLKEIKPLIDTCIKESKVEEETLGKLHN
GHEIPSSQSGKCFIACMAEHMKLMKDGKFEPAMTMEFIDKMVQDKAKADEI
KKAVDDCFKSVPDGDKCEMAASLATCMKEHHAELAGMN
>SfurOBP3
MKASASLTLIFLTLAVFHCSEAKIDKAKKEAAIKKCQAETSASDEDVKKVRKE
HVVPDSEEGKCFIACGFNHYDMLKDNRINLEGVNAFFEKLYDEQEKRDIAIKA
AASCAATESVSGLNDCHVAAKYFACLQRHPDFVKMKDDFDV
>SfurOBP4
MNTFQKFIFSGMVVLAGAMLITAEDTTIKIKNQQSPHKQQQVYCQAPPTAPER
LERIIEQCQDDIKTALLQEALNVLTDTSPRDLVKKTRSKREVFSGEEKRIAGCL
LQCVYRKVKAVDDQGMPTVPGLVRLYSEGVQDRNYYVATVQAVQQCVSASQ
HFRYYNPQVLKEDGYTCDLAYDMFNCVSDKIEAFCGRTP
>SfurOBP5
MVTSGLVQAVIAACLLLVTAYAYDFSDPYFNEHLQSAMEEIMEEEMLSIGRVQ
RDADQTQEVADEYFKCKHRNLKTCCGKINLMKNYGDKGKIYGKQCYEEVVS
AFKTNSSSTADDDDSMMDMFSCEKVKMIKLKHICVHECIGKKTKILKEDGSL
NAEEIKQYAREYMFNEEWSKELGERALDKCLTQSYNSVTKMLDEYEIKCNPT
SVQFHHCLWKEIEMTCPESKVDLKAKCVRLRERLRKQQAAGM
>SfurOBP8
MLYVLYFVIVTAASSAVITQIMAADSNNPDMQTVFNNCREEASATEDDIKTFR
AQQIPSTTTGKCMLACMFNHSGLMKEGKYNSEGALKLVGQVFAADPVKLGK
AKTLINTCSDEVKNENDKCEIASKIADCTVKMTSQVGLS
>SfurOBP9
MERTHVLIIAFAFIPFLSSAMQADFAMMQFPMQGTGTPMIQSIAGELKYCMDV
NAEQNSDGLEDYLPLLFNEELPTSLGQKCFLTCLFNRFGLLKDGFLDAQTAKT
LVETFYKDKHDEKTMANIAINVCRVSAVPDILNPCEIGFSLKSCFVDSNKKGKE
LRGKN
>SfurOBP10
MSTLLNFVFVFLVCLCSYSEAIPALTEAQIEQVGKAMANMCISSSGVQRSLITK
AMTGEIEDDRKLKCFFGCIMEAVQVTKNGKMQPEVLKRRANAMLPKTMRE
MILPTIDSCSHIENEDKCELAYSIVKCHFSVNGKNPFFFNF
>SfurOBP11
MLLEVCRFSVFLLALSATVYGRFSEEEKQLMNQVHTQCVTETGTSEDLVNKA
TNGDFAEDENLKCYVKCIWSTLTVMDDDGNFDVGVLEVMLPADMKDIVMK
AMSACIGAGGGSPCEKAFAVTKCLYKEAPADFFLP

>SfurOBP12
MRGYILVLCVLLFMRGMASGLVAAELEKLKNSCLKKSGATEDTARRLVGINV
VTENHVESCFLTCIYKGLKIVSSDNKFQPDTVKKIADDHFLGRNLKVTYQIAD
SCTKEIKADPADKCSIGASFRNCFSKYGKELGFFPHM
>TbraOBP1
MKTALSILAVCLVIAYVNGAAAPNNVKDKVGALAKKCAADHKANQEQAKIA
FTQKLPTDEVERCYLECVYTGVGVIQGGEYSVEGSKKLATQRFSDAKEHETV
NKLIDTCSKEVTKVKDEKCSLGRTVRECFVKHGEKVHFFPSAN
>TbraOBP2
MPKFYTSLLAFFIFSFFSMIIGNEEECMRQHNISRSFLDDEKREDKCFLACFMK
EQKIMNEEGHMVKEKMLEYFDQVEGDHPHAECREAVLHCVDMVEKEGDDC
ETAYKFDLCVNEKETVCALDEN
>TbraOBP3
MEARYCIEECVYKGVGLLDESSTELNHERLIQEFKRGVAGAGQWGTVMDEAI
NVCTGSSGQESSDSSCSEIPHAFTRCLIRQLFLNCPADKWNNSAECNLVKDRM
QVCPNIPPPPPIQHRPHNDSN
>TbraOBP5
MKAVTFLCLAASLLVIAQGESTTTEESATAQDNDKSTTVGGSTVSKTPEEIRQK
IKEQVEALTETCKTQAKLTPEQTKIATNMAVPKTEAEKCFLECIYNGIGLTKEG
AFYEQGARTLAQQRFMGAPDDLAKANTMIEACTKEVVVKDANEKCGLGRL
VRECFVKNGAKINFFPKP
>TbraOBP6
MGPSRIVVLVSTFIVSIMLLITDTHGAMTEAQMKQAMKTVRGMCLGKSGATK
EALDKMQEGIFDEEDRNLKCYLGCIMGMMQAVKNNKINLKMVRSQITKMLE
PEVGKRILTAFEGCQDTVGEDNCDLSFKFAKCLYDADPSAFIVP
>TbraOBP7
MNVIIDYENDRWFGQDHKRRQSGRGNGDSHSYGSNRREQNDENKYNSDPFN
RHYNTSRGGTTSIGSPLEDIDACVIHCIFRQMKMVSDESYLNRNTVLNVLTRRI
KDQELKAFIQEAINECFENLDPDDEEICDYSKSFAMCLEEKGKSNCDDWDLSA
KFDRNNRSRNNNNNNQNGFSYQNSRG
>TbraOBP8
MYQFHFLFAILVTINFVQSKVVHKRETHHEVLELKGLNKKELKDLELKVFEEC
RVESNLDKTTFDGYRKMENDVPNEEGFKKVSSCYSERMGFIANSKVNWEKL
KEAAEINHSDNKDYHDKSLNVIKDCEGKSYEELSHIDVAYKFAKCMKEGYLQ
AG
>TbraOBP9
MTLGHILIITGLIICAVTLVNSSNDVIKKNKEYAKNCMKKFPVKKSSLQEMYST
FHVPEDKNLKCFLGCLLRKVGLIKRNYIDWNVSRRAHKKLNQDPIVYKRTEV
MIKRCKKEIIPNFRDKCQLAADIISCKLKYSQKFGIPIMRMQ
>TbraOBP10
MMHIYAVLSLLSAITICKVDASGAETVKKALEVLSKCQKDFSVDDGTVTGIMS
FNGVPQSKEAKGMVTCWMNGVGFLKDGKPFVEEIKIWHQVLFKNEEHKKL
ADENVDFCVGNLKAKEESELAYELTKCFMTRAAETGLPPPELQIE
>TbraOBP11

MMYIYALLSLLSAITIHKVDASEGETVRKAMEVLSKCQKEFSIDNGTVIGFVSL
SDIPESKQAKGMVACWMNGLGFFKDGKPFVEGMKRWHKVMFRSEEHQKLA
DENAVICVANLKSEEESEMAYELCKCFVTRAKETGLPAPELRIE
>TbraOBP12
SAITICKVDASGAETVKKALEVLSKCFQKDFSVDDGTVTGIMSFNGVPQSKEA
KGMVTCWMNGVGFLKDGKPFVEEIKIWHQVLFKNEEHKKLADENVDFCVG
NLKAKEESELAYELTKCFMTRAAEVIIRKII
>TbraOBP13
MFLYTLFFVTHIVYMTNAVIDVGKLTTKSEVLETEKKTMEDCLNKAGLKYTM
LKDHLNGQTPEATKELKCVLGCYTEELGYVKDKKTQWHVIEMVHQIEYKTE
ENLEKGSQILKNCKTIVPEVAVDTCDAGYALYTCYMKQAIKVDLHP
>TbraOBP14
MFLYYLFLTHIIYITNAAVDIKGLTKAELAELEQKTMTECLKKHSVNETLLSEF
LNEETSESTSKEFKCVLGCYTEEMGYGKDKKPQWDVMEEVHKIEYDNDEDK
EKALKIVKTCKTIVPEEVEDSCELGFAMHSCYLDQSKKVGLLLV
>TbraOBP15
MMHPLIFVLGAVFFLMFDGAYAAVELSDDMKEMAKMLHDQCVDESGVNGA
LIEPCSRGDFADDGNLKCYFKCIFANMGALSDDGELDTDAFESILPPDLHDPLS
KMINNCKDPKGADACEVAFNFNKCLYTTDPEHFLVI
>TbraOBP18
MTRFIALTLFTLILVASITAQGNEKILQIFNKCSEMHKLSSDELAMIQSKESVPSS
PEAKCMTACMLKEGKLIIGSTYMKDNALMIADALFKDDASMAAKAREVVEH
CATEVGVDVGGDECEFAYKLAVCSDNHAKKISVTRPF
>TbraOBP19
EEVDFKGMTPKEIDDLEEKTYEDCRLELNISNSFLEEYLKTDTGVPDAIEFKK
QIACFNIKLGYVKGTKINWERIKLSFEVYYHDNKKKLDEDLKMVEDCQKMP
LDNLDEIEVSYLLAKCSKEGYAEVMECCLKNVN
>TbraOBP20
MKGFTTVCVFTIAITVSLAIDEAAKKKAINTFNKCKEQHPITDAELEQIKKHEG
LPSSQNAKCLAKCMLTEGNILKDGKYKTDIAIAFTETLHSDNAEEAEKARQV
VEHCASTVGTDVGSDACEYAYKMAQCGYSKAKEIGLEKPEWE
>TbraOBP21
MYMYSETGVINMKAELLCVLLTITIAKVISENTTVSDEDPETLQYINCIQKYNI
SGTEVPASGSYAEKCGNACAMKAKGMLTDEGEFIKDRMLSQKFQPYVTSEDI
VKMQMAIDFCVKQVSNEGDECEKAYGLMDCMQTKVMESSRSMK

The MEME analysis of CSPs
>BtabCSP1
ADTYTTQFDNIDLEAILKNEKLVDNYTKCLMDEGPCTNEGRTLKKLLPDALK
TACAKCTEKQKTGARKVIKFYQTQHPEDFKKLQQKYDPEGKFKAEFEKALFG
QTL
>BtabCSP2
APAEDKYTDKYDNINVDDILGSKRLLKSYLTCLLDKSPCTPEGSELKRLLPDA
LKTACSKCTEKQKEGAARIVERVTAEYPTEWKELSAKWDPTGEYWAKYKPL

VQEYLKASA
>BtabCSP3
AAATKESTKESTYTNKYDNIDLGKILTNDRLFLNYFKCLMDEHTCSPDGAELK
KVLPDALSNKCAKCTERQRSGSEKVIRHLIDNKPEMWAKLEAKYDPKGTYR
KTYKNEAEKLGIKV
>BtabCSP4
APAPLEQSDLEKFENMDLSSILSNKRLRTAYVNCMVDKGPCTADAAEFKKILP
DLTETQCADCSAKFKELIKKSVSTFQKDYPEDWKTLMAHFDPDNKRAADLE
KFMSS
>BtabCSP5
DDFYSDKYDNIDLDSILASKRLIRNYMNCFQGKSPCTPEGTYLNQVLPEALKT
ECAKCTEKQREGAVKAIKKLSAEYPEEWKEITDKLDPTGEQYAKFKARFP
>BtabCSP6
MVCSESIVRCLTYFQVISLVYRLASAQSNNRATYTTKYDYINVDAVMKNERIL
KMLVECMLERGRCTREGLELKAAVPDALATDCAKCSQMQRKHASRVIAYLIT
YKKEYWNALATKYDPDGSYRRKYGIPPQPQELSAGAAIQPSPNLIKPTKTVKK
TVTTVNNTNNLFKAKKRMTREEKKEMVRRRFPQLHFMPNMWASNIQKIEKK
SERAPSIARRNVRRKAHTPTRRHSTQRTAPKRGQETG
>BtabCSP7
MPAPQTTRATISDEALESALNDKRYLMRQLKCALGEGVCDPVGRRLKTFAPL
VLRGACPQCSPTETRQIQKVLSHIQRHHPKEWSKIVKQFTS
>BtabCSP8
ASTYTTKYDNIDLDEILNNDRIYKKYYDCLANKGKCTPDGKELKDILPDALKT
GCSKCNEKQKKGSEKVIKFLLDKKKADYDVLEKIYDSNGVYRQKYADEAKK
R
>BtabCSP9
APAEFYTSQFDNIDIESILKNEKLLDNYFNCLMDEGPCTLEGRTLKSLLPDALN
TSCAKCTEKQKKIARRVMTFYLDKYPANSARIIKKYDPENKFKDGIEKALLGS
R
>BtabCSP10
APAETYTTEFDGIDIDSVLKNEKLLDAYAKCLLDEGPCTREGRTLKTLLPDALE
TTCAKCSPTQKEKAKKVITFYMEKYPENAQKIMKKYDPTGKYRKALEEAFL
GSL
>BtabCSP11
AISDDEYRLETLCSSPALEHFDITPILKNDRLVSSYFKCFMDEGPCTNEGKMVK
RIIPEIMRTQCRNCNPTMRRIVRTVMKHMFQTRPRDVDDFFLKYDPHEMYYD
DLIEFMDEDNDY
>BtabCSP12
LPQKGPASTPRKQSVEEALGKKPEELPKTMKEALKRMEAVDVEKVLNNDRIL
TNYLKCFLNKGPCTSEAKNVKKSIALLVESRCVECDPKQRKIIKKSMQVVKTK
KPREYQELIKLYDPKGTQIAELEKFFASSK
>BtabCSP13
APPAGVDEKLLSKYDNFDVDRVLNNDRVLANYIKCLMDEGSCTNEGRDLKK
SIPDVLAGGCDKCTEKQKSVTEKVIKHLINKRPKDWDRLSKKYDPQGQYKN

KYADLYEKVQKEAAKESKEPSKPTKDTKETTKVTKDTKESAKAPKA
>AlinCSP1
MLKVLVLLAAVVCCVSAAATYTSKYDNIDLDEILSNTRLYKKYFDCLANKGK
CTPDGKELKESLPDALKTNCAKCTKKQQEGTDKVFRHVLKNKPNDYKVLESI
YDPPGIYRKKYEAEAEKRGIKLPGSH
>AlinCSP2
MKVAVLVLLCVGAALSAEVYTSKYDNIDVDKILSNDRILTRYIKCLMEEGNCT
NEGKELKKTLPDALASGCTKCSEKQKAQTEKVLRHLSKNRPRDWALLKTKY
DPKGEYSKKYEKEAKALTA
>AlinCSP3
MISKLSMVLLIGAFADVWAAEQYTDKYDNIDIDEILNNDRMYKNYFHCVMG
NGKCTPDGLELKAKIPEALQTECAKCTDKQKKEVEKVLRFIINQKKDDYKLL
EEKFDPEGVYRKKYEAQKKLVEEGKPIEY
>AlinCSP4
MRIILSAFLVAMACSLATCEMTEEEFYTKVFEEVDPDFILDNERILTSYLKCFYN
EIECNAHAEVVKKSIPDVLATVCGRCSDKQKSIFKYSLNKFIPAHPKDWEKILS
IYDPSGEAWPKVKAFIES
>AlinCSP5
MGHLTIVLLAAAFEVLTGSRAYTTHYDYIDVDQVLNNTRLYTKYVECLLGQG
KCTPEARELRDKLPEALQTNCARCSERQASESHRVIRFLIQNRQEDFKLLEAK
YDPSGLYFKRFEEETKRNVSLS
>AlinCSP6
MFYKLSVVVLMGILAGVWAADKYTDKYDNIDIDEILTNERLYKKYFDCIQGT
GKCTPDGIELKEKIPEALKTECAKCNEKQKAGVEKVMRYLITKKPEDFKILED
KFDPEGVYRKKYEAQRKLVEEGKPVEY
>AlinCSP7
MNYKLSVILLIGVLASVWAASTYTDKYDNIDLDEILTNERLYKKYFDCIQGKG
KCTPDGTELKEAIPDALKTECAKCNAKQKAGVEKVLRHLLTKKAEDYKILED
KFDPEGVYRKKYEAQKKLADEGKPIVL
>AlinCSP8
MDYKLSVMLLMGVLACAWAADKYTDKYDNIDIDEILNNERLYKKYFDCILG
NGKCTPDGTELKETIPDALKTACAKCNDKQKAGVEKVLRHLLTKKAEDYKIL
EAKFDPEGVYRKKYEAQKKLAEEGKPIAL
>AlinCSP9
LAVVTREMREREFFRQLEVINVDSILINQRLIDKYIKCLLKTGKCDPIMKDLRI
ALPLILGHLCEARCSEK
>AlinCSP10
MRSNFINESIPDVLATVCGRCSDKQKSIFKYSLNKFIPAHPKDWEKILSIYDPSG
EAWPKVKAFIES
>AlinCSP11
KFFFSGLLLVCMASVSLCADEYTDKYDSVDLDEILNNQRLYQKYIDCVMGKG
KCTPDGALLKEKIPEAS
>AsutCSP1
MLPFYVFSLCAVFVACQETYTSKYDNVNVEDALKNDRLYKAYFNCLADRGP

CTREGNMLKEALPDGLRNNCSLCTDPQRRGTHQVIRFLFKYRPEDMKLLEEI
YDPEGIYKTKYAEERKKLME
>AsutCSP2
MGHFPPVFSLSPVLLVASLHTMNTSTLLKIAFLLGCVAACLAAETRSSVSDEAL
EAALKDKRYLTRQLKCALGEGACDPVGRRLKTYAPLVLRGACPKCTPSEVRQ
IQQVLSHIQRHYPKEWAKILKQYAGQ
>AsutCSP3
MKFVAALLVASVAVLAVEAANQYTTKYDNIDLDDILKNQRLYKKYFECLTGK
GKCTPDGKELKEHLPDALKTGCSKCSEKQRAGSEKVIKHLLKNKPQDYAVLE
KIYDPSGIYKKKYEAEAKKLGINV
>AsutCSP4
MRIILSAFLVAMACSLATCEMTEEEFYTKVFEEVDPDFILDNERILTSYLKCFYS
EIECNAHAEVVKKSIPDVLATVCGRCSDKQKSIFKYSLNKFIPAHPKDWEKILS
IYDPSGEAWPKVKAFIES
>AsutCSP5
MDYKFFVVMQIGVISSVCAAGTYTDKYDNVNLDEVLNNERLYRNYFNCLQG
KGKCTLDGAILKEVIPSALKTDCALCSVRQKKGAEKVLIFLITKKPDDFKILED
KFDPEGVYRKKYEAQRKLVEEGKPIH
>AsutCSP6
MVCKLFAVVLMGILAGVWAADKYTDKYDNIDIDEILTNERLYKKYFDCIQGIG
KCTPDGIELKEKIPEALKTECAKCNEKQKAGVEKVMRYLITKKPEDFKILEDK
FDPEGVYRKKYEAQRKLVEEGKPVEY
>AsutCSP7
MVSKLSMVLLIGALADVWASELYTDKYDSIDIDEILNNDRMYKNYFNCVMG
NGKCTPDGTELKAKIPEALQTECAKCSDKQKKGVEKVLRFLIKEKKDDYKLL
EEKFDPEGVYRKKYEAQKKLVEEGKPIEY
>AsutCSP8
MDYKLSVMLVMGVLACAWAADMYTDQYDNIDIEEILTNERLYKKYFDCIIGN
GKCTPDGTELKETIPDALKTACAKCNDKQKAGVEKVLRHLLTKKAEDYKILE
AKFDPEGVYRKKYEAQKKLAEEGKPIVL
>AgosCSP1
MNILTIFCYVTVMCDTQVKPAVSAQRLQSVNQNVTPTNDGRKTIRETSSYPTR
YDYIDIEAVMNNERIIKILFNCVMSRGPCTREGLELKRIVPDAIQTECAKCNER
QRKQAGKVLAHLLQYKPEYWKMLVQKFDPNNVYLRKYMADNDDDEKLSL
QKLSNDTTKKKRNI
>AgosCSP2
MAHLNLFVVLIASLIYFTSAAEEKYTTKFDNFDVDKVLNNNRILTSYIKCLLDE
GNCTNEGRELKRVLPDALKTDCSKCTDVQKDRSEKVIKFLIKNRSTDFDRLTA
KYDPTGEYKKNLEKFEKERASAKPLKA
>AgosCSP4
MDSRIAVVCVVLAAFAVDQTVGAPQKDAVAASGPAYTTKYDHIDVDQVLASK
RLVNSYVQCLLDKKPCTPEGAELRKILPDALKTQCAKCNATQKNAALKVVDR
LQKDYDAEWKQLLDKWDPKREHFQKFQQFLAEEKKKGFTKF
>AgosCSP5

MHCKVLIALCCVAVYAVQASPAGTATAAAVSADDEIKDFPAYMKRFDKLNVE
QVLNNDRVLASHLKCFLNEGPCVQQSRDLKRVIPVIANNGCNGCTERQMTTI
KKSLNFLRTKKPTEWARLVKIYDPSGTKLNKFLDA
>AgosCSP6
MIKLILAIAFCVSITMTVVQTAPAKYTTKYDNVNIDEILNNDRLVASYFKCLME
TGKCTPEGEEIKRWLPEAIENKCEDCSEKQKLGSEKIIKFLFEKKNDMWKQLE
AKYDPQGTYRQRYAEEAKKLNINV
>AgosCSP7
MSRSSSSVTMKVFVIAICVCAALARPEDVKVENKPAVIKSETLAAPLPTNIVKR
ATDTIQLDSSLPNVSEDVLDKALSDRRFVQRQLKCATGEGPCDPIGRKIKAHA
PLVLRGMCVKCSQSEIKQIQRVMSHIQKNYPKEYTKMLKQYQSGF
>AgosCSP8
MNNIIMNNSRGRYGIFSLLAVTIAAIMLVHQPATVRCADGGIITPQQQQQQTM
MFTAPTGYYVSTYDHIDVGRLLRNNKVVSGYVKCFVNEGPCTPDGKLVKAY
LLPEIIRTVCGKCTPRQKDMARMVLKHIYTYRQADFEKIMQIYDTDGKRNEIL
AFMNH
>AgosCSP9
MSAFCLNSFILMTMITVIVTHATFTRSTKFDDRTGIDIHLVKRDTDDVNDDENS
VESDEGFFYRFTHFFQDSSDKEDDDDDEKKPDFITTFDIFKLLDEEYAMQQFY
CVINEDPCDEVGMRLKATIPEEINRNCERCTSTERNNIRRILNYVKKHYPQFW
KRVEPIYKKKI
>AgosCSP10
MINTRPRKLVRCIRGVSISVAKGDDAVNAENKDDDSHLVNREEIQRYMSMME
KINIDQMLNNTRLMSNNVKCFLNEGPCTAHLREMKKMVPMLVKDSCSSCTK
EQKIMMKKAMDAVKARRPNDYEKLSKFFDPEGKYEKKFLENLNESK
>AlucCSP1
MLKVLVLLAAVVCCVSAAATYTSKYDNIDLDEILSNTRLYKKYFDCLANKGK
CTPDGKELKESLPDALKTNCAKCTKKQQEGTDKVLRHVLKNKPNDYKVLESI
YDPTGIYRKKYEIEAEKRGIKLPGSH
>AlucCSP2
MASKLSVVLLIGAVGMVLAADKYTDKYDNIDLDEILGNQRLYQKYFDCIQGK
GKCTPDGAELKETIPEALKTECAKCSDKQKAGVEKVLRHLIREKPDDYKVLE
DQFDPEGVYRKKYEDLKKKVEEGKPIEY
>AlucCSP3
MKVAVLVLLCVGAALSAEVYTSKYDNIDVDKILSNDRILTQYIKCLMEEGNCT
NEGKELKKTLPDALASGCTKCSEKQKAQTEKVLRHLSKNRPRDWNRLKNKY
DPKGEYSKKYEKEAKAISA
>AlucCSP4
MKFVAALFVASVAVLAVEAADQYTTKYDNIDLDDILKNQRLYKKYFECLTNK
GKCTPDGKELKEHLPDALKTGCSKCSEKQRAGSEKVIKHLLKNKPSDYAILEK
IYDPQGSYKKKYEAEAKKLGINV
>AlucCSP5
MVGKLSVVLLIGAVGMVLAAELYTDKYDNIDVDEILGNQRLYQKYFDCIQGK
GKCTPDGAELKKNIPEALQTDCAKCSEKQKAGVEKVLRHLINEKPEDYKVLE

EQFDPEGVYRKKYEHLKKKVEEGKPV
>AlucCSP6
MVSKLSIVLLIGALADVWASELYTDKYDNIDVDEILGNQRLYQKYFDCIQGKG
KCTPDGAELKKNIPEALQTDCAKCSEKQKAGVEKVLRHLINEKPEDYKVLEE
QFDPEGVYRKKYEHLKKKVEEGKPIEY
>AlucCSP7
MVSKLSIVLLLGALADVWAAELYTDKYDNIDIDEILNNDRMYKNYFNCVMG
NGKCTPDGLELKAKIPEALQTECAKCSDKQKKGAEKVLRFIINQKKDDYKLL
EEKFDPEGVYRKKYEAQKKLAEEGKPIEY
>AlucCSP8
MLKVLVLLANAASTYTTKYDNIDLDEILSNQRLYKKYYDCLANKGKCTPDG
KELKEALPDALKTNCSKCSKKQQEGTDKVLRYVLKNKPNDYKVLENIYDPS
GNYRKRYEDEASKRGIKLPGSH
>LeryCSP
MDSRIAVVCVVLTVFAVDQTVGAPQKDTAVVNGPAYTTKYDNIDIDQVLASK
RLVNSYVQCLLDKKPCTPEGAELRKILPDALKTQCTKCNATQKNAALKVVDR
LQRDYDKEWKQLLDKWDPKREYFQKFQQYLAEEKKKGVVKF
>MperCSP1
MNTLLLAVALCIAITMTVVQTAPAKYTTKYDNVNIDDILNNDRLVASYFKCLM
ETGKCTPEGEEIKRWLPEAIENKCENCSEKQKIGSEKIIKFLIEKKNDMWKQLE
QKYDPQGLYKQRYSEEAKKLNLDV
>MperCSP4
MTNNNMNSPRCRPEIFSLLAVAAIATVLVHQPSTVHCADAGVYPPQQQQQEAT
MFTAPSGYYVSTYDHMDVGRLLRNNKVVAGFVKCFTNEGPCTPEGRLAKAY
LLPEIIRTVCGKCTPRQKDMARLVIRHIYTYRRGDFDKIMQIYDTDGKKNEIID
FMNQK
>MperCSP5
MNCKVLIALCCVAVYAAHASPAGAATAAAASADEEIKDFPAYMKRFDKLNVE
QVLNNDRVLASHLKCFLNEGPCVQQSRDLKRVIPVIANNGCNGCTERQMTTI
KKSLNFLRTKKPVEWARLVKIYDPSGTKLNKFLDA
>NlugCSP1
MFKNVLLVCLLVAVVSAKPKPAEKKQYTTKYDNIDLDEILNNQRLFDNYYKC
LLGGKCTPDGQELREALPDALATACSKCTEKQRVGTEKVIKYLIEKKPTEYSE
LEKKYDPQGNYKRKYQAEAAKRGIKV
>NlugCSP2
MSKLPVTLVLMLAVFSVDCGKLYKDRYTTKFDKIDLDEALNNQRLFESYLKC
LMGDKCSPDGYELREALPDALATACAKCSEAQKAGTEKVIRFLIEKRPKEYAL
LEKKYDPEGIYRDKYKPIAEMKGIKLD
>NlugCSP3
MKFLCVTIFECALIVVAFGMPQDTTYPTTYDDVNVDDILHNDRLFNRYFTCLT
KKEGCTPEGKLLAATIPDALATTCAKCSAKQKTAAEKVIKYLYFNKRDKFDEL
AKIYDPESNYLNKYLVDGFPAKV
>NlugCSP4
MFLIAVWALSPRRLPWGLPWGGLAGVAAQQQAKNTRYTTRFDSIDVEVILKN

ERIFRRYMDCLLDKGRCTPEARELKRLLPEALKTECLKCSEVQRRQGAKVMA
FIIKNKRPSWELLLAKYDPQGIFRAKYMYNENNIEAVLKQLEREQQGIYGTYS
STNSTTSSNSTSIR
>NlugCSP5
MRCLLLVAVVCAALVAVCHAQDSKYTSKYDNIDIDKILKNDRVLSQYIKCLMG
EGSCTQEGRELKRLLPDAIQSNCSKCSEKQRSASVKVMRHLRQSRERDWNRL
LDKYDPQGDKRKNLKLD
>NlugCSP6
MLWAARFIVLPLLFCVLQVWSAPADEKYTDIDFDSILANRRVLSSYVKCLTDK
GPCTPQGKELKKIVPEVIQTSCTKCSPQQKKVVRNVITTMQSKYKDQWDLVV
NKYDPKKQRSGELKAFLSGTD
>NlugCSP7
MASASSGTTSTTSAPKTAESASAKSSSKDEIPDQTFDRYINNERYMLMQYECL
MGNKPCDHVGRKLKAAVPLVVRGLGCPKCSQREEDQMKRIVSHVQRSYPDK
WQKLIKKYGN
>NlugCSP8
MSSTMLVFVAVLCFSAVLAKPADKYTTKYDNIDLDEVLSNQRLFDSYFKCLM
GGKCTPDGQELRDALPDALATACEKCSEKQKEGTEKVMKFLIEKKPTEFAELE
KKYDPQGTYRQKYKAEADKRGYSV
>NlugCSP9
MKSQQLLVSCLFICTWLVVLMAPSANAAPKEKDPERKALYRLEYIDIEKVLDN
NRMLTNFIRCFLRQGPCTPEARDFRKLLPKLAKTMCSDCTARQRYIIKKVFKH
LMEERPKEWELLMDRFDPQRKYAERLDTFMVDMTTRAPVTSSPMPSSPVTLT
SSSVTMSSTTQRVIEILRTSTDMSNESRPAS
>NlugCSP10
MFMLLACSELGSGQQQQNVDNIEMSIYDKMFENMDVNSLLKNHRLVDSYLK
CFLNEGSCTHIGHEVKMMIPEVIKSRCGTCGENQMRALKAGLRLFIVLRPDD
WQRFLDVYDPDRKEWPHIKAFMDSDD
>PsolCSP1
MPSVLTTVLPSLLNRWLEMPLSVLLCVVDLCFLTANDGNSTIDITRASTMTDR
LDAVDIDEVLKNTRLFNSYVKCVLETGPCTAEGREMRREMLEVLQTGCANCT
EKTKVRIKKSFLRLKSDPRFRESYKKVLDKYDPDKKYIANLEKFLT
>PsolCSP2
MKLLVVLFTIAFAIILSFSLCKGEANAANNNDIDSLLADKNFVRRQIHCVLGKA
RCDKFGNNLKASIPKVISQNCQSCTPEEAANANKIVSFVKQNYPDVWKKVAQ
RYSGQ
>PsolCSP3
MVLACSAALLLVAVEAADYTNKYDGIDVDKILQNNRILNNYIKCMLDEVTCT
AEGRELKKVLPDALATGCTKCNDKQKETAQKVITHLMDKRPNDWERLVKKY
DPKGEFKKRFEAQGKKF
>PsolCSP4
MKSFTFATLFLIAFAFTLAEDKYTSKYDNFDVDSVLNNDRILTNYIKCLLGEGP
CTNEGRELKKVLPDALSTGCNKCNDKQKSVSRKVLVHLIEKKPAEYKKLAKK
FDPDGKFEEKYKADILNATEKPKA

>PsolCSP5
MKNLVVLTLFLAVAGVAYAADTYTTKFDNIDIDQILHNERLLKPYVNCLLTDT
KCTADANELKRVLPEALVTNCQKCSEKQKQGAEKVISYLAKNKPEIWSKILA
KYDKNNEYRTKYAETARRLGVPL
>PsolCSP6
MSAALWLLFFCLLNMCLAADLPNAQPTYTTRYDYLDIDKILTNDRILKRLMD
CIMDRGPCTREGKELKRIIPDALKTNCSKCNDRQKMNTGRALAYLLHYKPEY
WFELLNRFDQDGSFRRKLNIDADDSSERSSAIVRKTRSIFLNRFRIEPSKKKLL
NAGA
>PsolCSP7
MNQFVSFVALFLSLVSQIMCTTPATSTEDNSKQVDDVVRNEKIREVYIKCLLD
KGPCSNEAADVKKMLPEAVQRGCEKCSEMHKKIIEKMMIFLKERQPETLQQIT
AKFDPKGEFMKTFLTKLQQQHNQSNESGQQYGQNAPVFNQGQNDRNHQQA
KPVQN
>PsolCSP8
MKSFSIFAIALCAVVCCSVADDTYTTRYDNINLDEILASKRLVANYVQCLVNGK
PCSPEGTELRKILPEALKTKCAKCTPKQKEGAIKVITRVQKDYPEEWKKLAAK
WDPTGEYFRAFSAEAQAQA
>PsolCSP9
MSRRLSFFLLALCALAALAATEEIYSDKYDYINIEEILANPGIRARYYNCFLET
GPCQTPDAKFFKEKFPEALVTKCRKCTEKQKVSFQKIVEYYTEKEPEKWKMV
LTKAINDAQKKRSI
>PsolCSP10
MNKLVLLALCALAVSVAYAEEEKFTDKYDNVDLDEVLKNERLFNSYYKCLM
DQGKCTAEGAELKKYLPEAIKTECAPCTEKQKEGAKKVIKHLTKNKPTEWKN
LMDKWDPERKYREKYESRIDEILNS
>PsolCSP11
MTSSVIYLFALSASIVVSVTCHKTLVESILEKYKADCEKVTDMEPFLKNEKLVT
THVKCLLGEIRCNLDGERIKKAILEVLPDACAKCTEKQKTIVRQVMQYMYKN
RQSDFDKIFKIFDPDMKYKEKMMDFMNKKE
>PsolCSP12
MKVSTFKMDSKIISVFILISVIVTLSYAGEEEIKTIGDFLKKFGNINMEEVLQND
RLFQAYHKCFLEQGPCTPEAREMRNVIPSLVKTACDACTPEQKKEMKKHLDY
ARHNRAKEWEELLDKYDPKREILEKFLSSVPDK
>SaveCSP1
MAQLNLFVVLVASLVCFTLAEEKYSTKYENFDVDKVLNDDSLLTSYINCLLDE
GNCTEEGQALKRVLPDALKTNCGKCTDTQKMKIEKILKFLMKNRSTDFDRLT
AKYDPSGEYKKKLEKFSA
>SaveCSP2
MDSRIAVVCVVLAVFAVDQTVGAPQKDALAAGSPTTYTNKYDHIDIDQVLAS
KRLVNSYVQCLLDKKPCTPEGAELRKILPDALKTQCAKCSATQKNAALKVVD
RLQKDYDKEWKQLLDKWDPKREQFQKFQQFLTEEKKKGVVKF
>SfurCSP1
MFNLLTLVVCLSTIAVQIQAAPEEAQYTTKYDKINLDEILNNDRLFKSYFGCL

MGGKCTPDGQTLRDILPDALETACSKCSDTQKAGTEKVFKFMIEKKPSEFADL
EKKYDPNGKYRARYEADAEKFGIKV
>SfurCSP2
MVLADTPTTSPKVETKAVESGKSSSKDEIPDQTFDRYINNERYMLMQYECLM
GNKPCDHVGRKLKAAVPLVVRGLGCPKCSQREEDQMKRIVSHVQRSYPDKW
QKLIKKYGN
>SfurCSP3
MQLLYALVFGCTLVMVSSDMPQSTYPTKYDDYNPDDILKNDRLFNQYFICLT
KKKGCTTAGELLSAIIPDALATSCAKCSAKQKAIGEKVIRFLYFNKPDEFAEMS
KIYDPEGKYLEMYIASGGLI
>SfurCSP4
MKCPLLSVSCLWISLLALSSSASAATKEKDPERKALYRLEYIDIEKVLDNNRML
TNFIRCFLRKGPCSPEARDFRKLLPKLAKTMCSDCSPRQRFIIKKVFKHLMEER
PKEWELLMDRFDPQRKYAERLDTFMVDMTTPSTTTTTTSTTPSTPMSSTTQRII
EILRTSTEMSNESSP
>SfurCSP5
MSEILVTSLIFMLLAASELGLGQQQQTQKPQQQNVDNIEMSIYDKMFENMDV
NSLLKNHRLVDSYLKCFLNEGSCTHIGHEVKMMIPEVIRSRCATCGENQMRA
LKAGLRLFIVRRPDDWKRFLDVYDPDRTEWPHIKAFMESDD
>SfurCSP6
MKLALFCCLLGLVAAVSAQSEKSEKPEKYTTKYDYINVDEILSNDRLFNSYYK
CLMGGKCTPGGPELRTHLPDALQTNCSKCSEKQKEFSDKVIKHLMDNKPEEF
SALVKKYDPEGIYKDAFKPKHNQ
>SfurCSP7
MRASKASSLVAVLLIAVWGFTGVQAQQKSKDTRYTTRFDSIDVEVILKNERIF
KRYMDCLLDKGRCTPEARELKRLLPEALKTECLKCSEVQRRQGAKVMGFIIK
NKRPYWDLLLAKYDPQGIFRAKYNYNENNIEGVLKQLEREQQGLYGTYSNT
TNTTNTVNSTSTRK
>SfurCSP8
MLKFTLTLLVLAVVSVNCGKLYKDRYTTKFDKIDLDEALNNQRLFESYLKCL
MGDKCSPDGYELREALPDALATACAKCSDAQKAGTEKVIRFLIEKRPKEYALL
EKEYDPEGIYRDKYKPIAQEKGIKI
>SfurCSP9
MRCLLLVAVVFAAFIAAARADEANKYTSKYDNIDIDKILKNDRVLSQYIKCLM
GEGSCTQEGRELKRLLPDAIQSNCSKCSEKQRQASVKVMRHLRQSKERDWN
RLLDKYDPQGDKRKNLKLD
>TbraCSP1
MRSMLTVCLLCMFVAACYTSSKYTTSYDNLDLDEILNNHRLYTRYFQCLTNK
ARCTPDGKELKAVLPDALATGCAKCTEKQKQGSEKVIRFLLKNKPGDYEELE
KMYDPNGTYRHRYEEEAKKLGIKV
>TbraCSP2
MSRTIAFFCLFVVACAYATSTYTTQYDNIDLDEILNNDRIYKKYFDCLAHNIKC
TPDGKQLRDILPDALKTACSKCSEKQKKGSEKVIKFMLEKKRADFDVLESQF
DPTGIYRKKYAEEAKKHGVHI

>TbraCSP3
MSRTIAFFCLFVLVCTYAASTYTTKYDNIDVEEILNNDRLYKKYYDCLANKAN
CTPDGKELKDILPDALKTQCAKCSEKQKKGAEKVVRFMLENKRADFDALEKI
YDPSGTYRKKYAAEAKRRGINV
>TbraCSP4
MTRTIAFFGLFVVAGAYAASTYTTQYDNIDLDEILNHDRVYKKYFDCLVNKAK
CTPDGKELKDILPDALKTGCTKCNEKQKKGAEKVIRFLLENKRADFDALEKI
YDPSGTYRKKYAPEAQKYGINV
>TbraCSP5
MQGLTVVLICSLATALSATTYTTKYDNIDLDAVLRNDRVYKTYYNCLTNKGK
CSPEGKELKDKLPDALQTGCSKCSARQKQGLEKIIKFLMNSKQADFKEFERIY
DPTGEYRKRYAEQAKKKGIKV
>TbraCSP6
MYAKLWIILAITVYICHAAKQSKVTYTDKYDKIDVDAILHNERVLKRYIDCLM
DRARCTPDGAELKKYIPEALETECAKCTEAQKRFAGKVMSFLLQNKRNYWN
ELLGKYDPNGKFRKKYEEAALQDEDYSSFYNE
>TbraCSP7
MKASLLLLQVSIVLVAMVTWNMAAEGRSAVSDEALEAALKDKRYLARQLKC
ALGEGACDPVGRRLKTYAPLVLRGACPKCTPSEVKQIQQVLAHIQRHYPKEW
SKILKQYAGQ
>TbraCSP8
MLFRHHTLSAIVLFFSLNLASTLTLQQIHRSWFQRLSVIPVEDVLNNRRILNKY
LGCLLRRTVCAPEARDFRILLPSILREPCNNCTERQRSSLKKIFEHVHTVHPGE
WQEIMSMYDPKSEHQEKIINFISNS
>TbraCSP9
MKVITIVLICLSAFVVTIYAQYEVNVEEILNNKRLLDAYTKCYLDRGPCPGPAR
ESKKKLGEVFTTNCAKCNKKQRQDTRAALRKLRERKPQLFLEIFEKYDAGSK
HLDGFLIWLKKND
>TbraCSP10
MKKMKLLKLVALLILSASISTAEEPDNEFNRLFDPNVDVDAVLDNDRVLNAYL
ACFYDEGPCAERPKLVKSKIREVLETTCGKCNDQQRQRLKYILNKFIDKRPND
WQRILEIYDPDGAFRDNVEKLKQGLPAQ
>TbraCSP11
MMLLSLCLAWLMFGMAFCRPDSEEDAFYYQVFEEIDVDIILDNERLLRSYLTC
FFDEAPCSAHAAAVKESIPEVMSTVCGKCNDKQKAIYKHALNKFIPTHKEDW
DHILRIYDPNGEYWPNIKKFMES
>TbraCSP12
MKSAAIIVFLLVICAGTNLASTYTTKYDNIDLDEILSNERIYVKYYNCLLNKSR
CTPDGKELKENLPDALQTACDKCSEKQKQGSEKVLRFILEHRPQDYLALEEM
YDPQKVYRHKYEKDAKERGLKFPD
>TbraCSP13
MMSATILAFSTIIAASFAASTYTTQYDNIDLDEILNNERVYIKYFNCLIGKDQCT
PDGKELKETLPDALKTECSKCTEKQKAGAEKVLRFVIENRPDDYKQVEAIYD
PEGIYRQKFGAEAKEKGIELPK

>TbraCSP14
MKCTVVFFIVATFGAVICDEGTYTTKFDNIDLDEILKNERIYEKYLLCLKDEGK
CTPDGRDLKDSLSDALQSGCEKCSEKQKAGTQKVLRFVLEKKPNDYLALEKI
YDPNRIYRIKYKDEAEKLGLKFPNK
>TbraCSP15
MKCTVVFFIATIFGAVICDEGKYTTKYDNIDLDEILNNDRIYEKYFLCLKGEGK
CTPDGRELKVALPDALKTRCVKCSEKQRAGTEKVLRFILDKKPNDYLVLEKIY
DPDRMYRVMYKEDAEKLGLKFPTK
>TbraCSP16
MPLVFIGSIAAGEVYTSKYDNIDVDKILSNDRILSQYIKCLMDEGNCTNEGKEL
KKTLPDALATGCNKCSEKQRAQTEKVLRHLIKNRSRDWARLKGKYDPTGEY
SKKYETKVPASK
